# Monounsaturated and polyunsaturated fatty acids concerning prediabetes and type 2 diabetes mellitus risk among participants in the National Health and Nutrition Examination Surveys (NHANES) from 2005 to March 2020

**DOI:** 10.3389/fnut.2023.1284800

**Published:** 2023-11-24

**Authors:** Susu Jiang, Wenhan Yang, Yanmei Li, Jingying Feng, Junjie Miao, Hongmei Shi, Hongmei Xue

**Affiliations:** ^1^Department of Child and Adolescent Health, School of Public Health, Guangdong Pharmaceutical University, Guangzhou, Guangdong, China; ^2^Food Science School, Guangdong Pharmaceutical University, Zhongshan, Guangdong, China; ^3^School of Public Health, Hebei Medical University, Shijiazhuang, Hebei, China; ^4^The First Hospital of Hebei Medical University, Shijiahzunag, Hebei, China; ^5^College of Public Health, Hebei University, Baoding, Hebei, China

**Keywords:** prediabetes, type 2 diabetes, monounsaturated fatty acids, polyunsaturated fatty acid, NHANES

## Abstract

**Objective:**

Unsaturated fatty acids (UFA) may be related to glycometabolism. While associations between UFA intake (especially their subtype) and prediabetes or type 2 diabetes mellitus (T2DM) need to be further studied. In this study, we aimed to evaluate the potential relation of UFA with prediabetes and T2DM.

**Methods:**

A total of 16,290 adults aged older than 18 years from the National Health and Nutrition Examination Survey (NHANES) from 2005 to March 2020 were included in the present analysis. Dietary intake was assessed by two day, 24-hour dietary recalls and daily intake of total monounsaturated fatty acids (MUFA) and polyunsaturated fatty acids (PUFA); four specific fatty acids of MUFA and seven specific fatty acids of PUFA were calculated. Prediabetes and T2DM were diagnosed by fasting glucose, glycohemoglobin, and self-reported medication or insulin. Rao–Scott modified chi-square tests, the Taylor series linearization method, and multivariable logistic regression analyses were applied to analyze the associations of dietary MUFA and PUFA intake with diabetes risk.

**Results:**

Of the participants, 44.34% had prediabetes and 13.16% had T2DM patients. From multivariate analysis, we found that intake of MUFA, PUFA, and some subtypes was negatively associated with the risk of prediabetes and T2DM in Americans. Compared with adults in the lowest tertile, those in the highest MUFA (PUFA) tertile had an approximately 50% (49%) and 69% (68%) lower risk of prediabetes and T2DM, respectively. Moreover, the effects of the subtypes of MUFA and PUFA on prediabetes and T2DM were different. Higher intakes of MFA 18:1, MFA 20:1, PFA 18:2, and PFA 18:3 and higher tertile intakes of MFA 16:1 and PFA 20:4 were related to a lower risk of prediabetes and T2DM. Similarly, the effects of MUFA, PUFA, and subtype on prediabetes and T2DM varied among different age groups, being weakened along with age.

**Conclusion:**

Our study suggested that total MUFA and PUFA intake might be essential in preventing prediabetes and T2DM, especially in Americans. However, this protective effect may decrease with age. Moreover, the effects of the specific UFA on prediabetes and T2DM need further consideration.

## Introduction

Type 2 diabetes mellitus (T2DM), becoming one of the leading causes of death worldwide and a major public health problem, places a heavy and growing financial burden on healthcare systems in many countries (1). Moreover, the prevalence of T2DM has risen rapidly over the past decades and is projected to continue to rise. Data from the International Diabetes Federation (IDF) show that ~537 million adults (20–79 years) were living with diabetes in 2021 (2). Additionally, the total number of people living with diabetes is projected to rise to 643 million by 2030 and 783 million by 2045 (2). Furthermore, the prevalence of prediabetes (a high-risk state for diabetes defined by glycemic variables that are higher than normal but lower than diabetes thresholds) is increasing worldwide (3). Notably, individuals with this condition are at increased risk of developing T2DM and other chronic diseases. Therefore, we cannot ignore prediabetes while focusing on diabetes.

Dietary intake is a crucial and modifiable intervention factor for diabetes (4). Fatty acids have been reported to regulate gene expression by modifying epigenetic mechanisms, resulting in positive or negative impacts on metabolic outcomes such as T2DM (5). This effect might result from its fundamental effects on insulin transduction signals, insulin sensitivity, oxidative stress, and glycemic control (6).

A high intake of unsaturated fatty acids (UFA), including polyunsaturated fatty acids (PUFA) and monounsaturated fatty acids (MUFA), has been shown to reduce the risk of T2DM. Mediterranean diets rich in PUFA and MUFA have been reported to be protective against the risk of diabetes (7–9). Moreover, the IDF (2) suggested a healthy lifestyle by replacing saturated and unsaturated fats. It is noteworthy that whether dietary fat quality, especially the intake of specific types of UFA, is associated with T2DM is controversial, as revealed by systematic reviews and meta-analyses published in recent years. Zhou et al. (10), Chen et al. (11), and Qian et al. (12) performed a meta-analysis to investigate this association from cohort studies and suggested that the intake of n-3 fatty acids might be weakly positively (10), invertedly *U*-shaped (11) or negatively (12) associated with the T2DM risk. A systematic review and meta-analysis of randomized controlled trials showed that increasing omega-3, omega-6, or total PUFA has little or no effect on preventing and treating T2DM (13). Hu et al. (14) also conducted a dose–response meta-analysis of cohort studies that reported non-linearly significant associations between specific PUFA intakes and T2DM. Thus, large-scale population studies are needed. However, large-scale studies on the relationship between PUFA and MUFA are limited.

Considering the limited research and urgency to solve the scientific problem, we pooled seven cycles of data from the National Health and Nutrition Examination Survey (NHANES), a complex, multistage probability sample of US civilians, to examine the relationships of total MUFA and PUFA intake to prediabetes and T2DM risk and evaluate the potential ability of subtypes of MUFA and PUFA intake. Simultaneously, we performed a sub-analysis to observe the age difference in their relationship. The results may provide data support and ideas for using UFA in diabetes prevention and control.

## Materials and methods

### Data source and study population

We used data from NHANES, implemented by the National Center for Health Statistics (NCHS) (15). Details of the survey are described on the CDC website. In brief, NHANES combines personal interviews with standardized physical examinations, laboratory tests, and 2-day food frequency reviews, administered by specially trained staff who travel to selected survey sites to collect data on a nationally representative sample of Americans (16). The NCHS Ethics Review Board approved this study, and informed consent was obtained from every participant (17).

**Figure 1:**
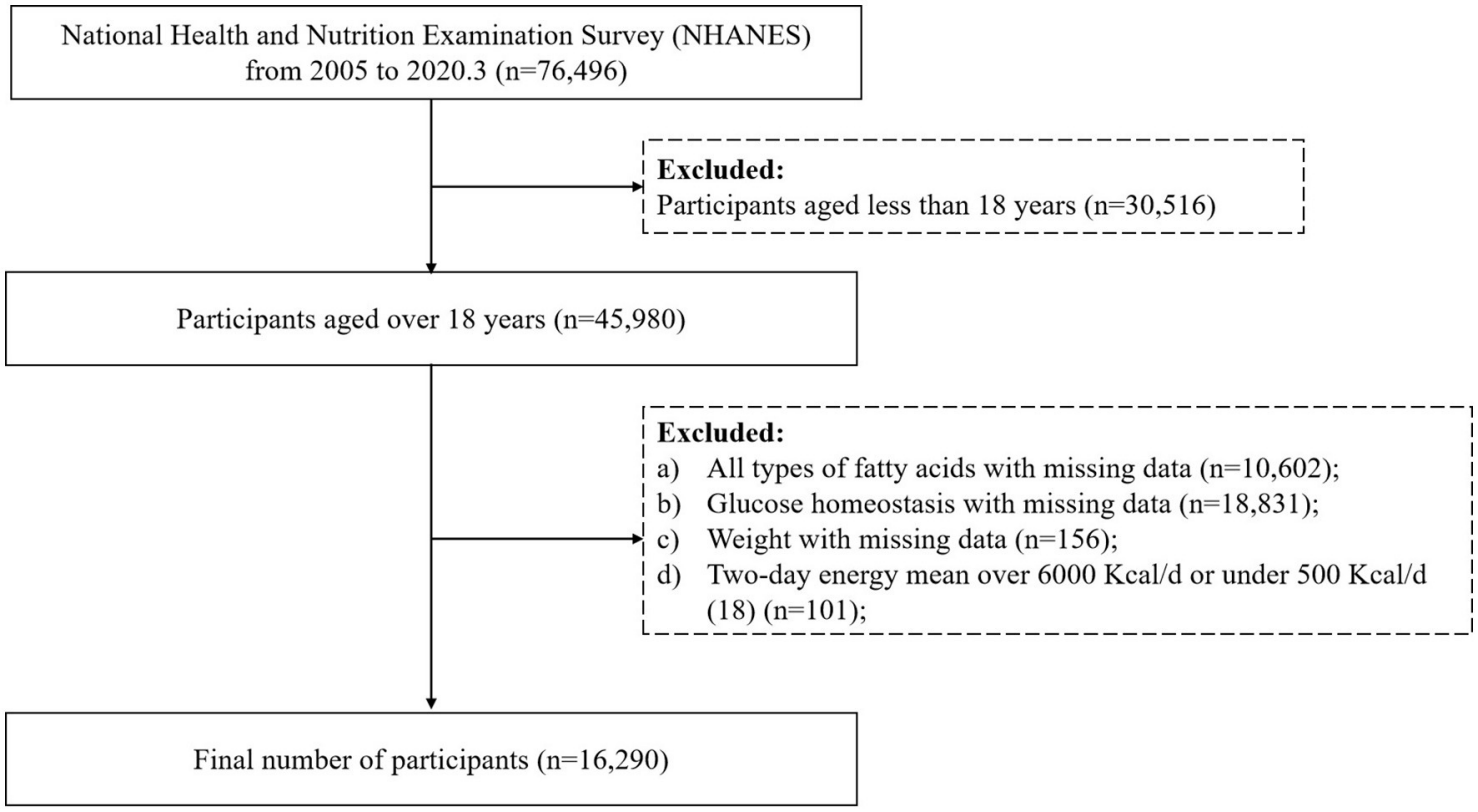
Flow chart of the study sample.

Publicly available data from NHANES 2005–2006, 2007–2008, 2009–2010, 2011–2012, 2013–2014, 2015–2016, and 2017–2020 were used in the present analysis. From 2005 to March 2020, 76,496 individuals were recruited. Of those, 45,980 aged older than 18 years were included in this analysis. Then, any person who met the following criteria was excluded: (a) any person whose energy intake and all types of fatty acids on dietary consumption frequency were missing (*n* = 10,602); (b) those without information on fasting glucose level and glycohemoglobin A1c (HbA1c) (*n* = 18,831); (c) those with a missing weight value (*n* = 156); (d) participants with a daily calorie intake of < 500 Kcal/d or above 6,000 Kcal/d (18) (*n* = 101). Finally, 16,290 people were included in this cross-sectional analysis. The flow chart is presented in Figure 1.

### Fasting blood glucose and definition of diabetes

The fasting blood glucose is tested according to the Mobile Examination Center (MEC) laboratory procedure manual. Before testing, participants were asked to complete the fasting questionnaire file, which included auxiliary information such as fasting status, length of fasting, and the time of venipuncture, assisted by trained staff. If participants provided blood specimens but did not meet the 8–24 hour fasting criteria, their sample weight value was assigned as “0”. Blood was processed and aliquoted into vials. The vials were then refrigerated or frozen (−30°C) before being transported to testing laboratories for analysis (19).

Definition of T2DM and prediabetes was according to the following criteria (20): (a) participants whose fasting glucose was ≥126 mg/dL, HbA1c was ≥6.5%, or taking medication or insulin were diagnosed as diabetic; (b) participants whose fasting glucose was 100 to ≤ 125 mg/dL, HbA1c was 5.7 to ≤ 6.4%, and not taking medication or insulin were diagnosed as prediabetic.

### Dietary intake assessment

Participants who completed a 24-hour dietary recall (first day) interview during their health examination in the MEC were then asked to complete a second 24-hour dietary recall (second day) interview after 3–10 days of the first recall. Recall interviews were conducted in person and over the phone. Upon the completion of the in-person interview, participants were given measuring cups, spoons, a ruler, and a food model booklet, which contained two-dimensional drawings of various measuring guides available in the MEC to report food amounts during the telephone interview. The energy intake, MUFA, and PUFA were collected through the in-person MEC by using a set of measuring guides (measuring cups, spoons, a ruler, and a food model booklet), which was available to use. There were four specific fatty acids in MUFA and seven specific fatty acids in PUFA. MUFA included MFA 16:1 (hexadecenoic acid), MFA 18:1 (octadecenoic acid), MFA 20:1 (eicosenoic acid), and MFA 22:1 (docosenoic acid). PUFA includes PFA 18:2 (octadecadienoic acid), PFA 18:3 (octadecatrienoic acid), PFA 18:4 (octadecatetraenoic acid), PFA 20:4 (eicosatetraenoic acid), PFA 20:5 (eicosapentaenoic acid, EPA), PFA 22:5 (docosapentaenoic acid, DPA), and PFA 22:6 (docosahexaenoic acid, DHA). The daily dietary energy intake, UFA, and subtype intakes were calculated using the Dietary Research Food and Nutrition Database for Dietary Studies of the US Department of Agriculture (21). Furthermore, total MUFA and total PUFA intake were calculated by the average intake for 2 days. All types of fatty acid intake were adjusted by body weight (mg/kg/day) before being put into the multivariable model.

### Covariates

Information on sociodemographic, physical, and biochemical indexes was collected as covariates. Sociodemographic variables included age (18–39 years, 40–59 years, and 60 years and older), sex (male and female), ethnicity (non-Hispanic white, non-Hispanic black, Mexican American, and others), education level (less than a high school graduate/GED, high school graduate/GED, or above), and family income–poverty ratio (< 1.30, 1.30 to < 3.50, and 3.50 and over). Trained health technicians in the MEC measured anthropometric variables, such as height and weight.

### Statistical analysis

The study accounted for appropriate survey design factors in statistical analyses, such as sampling weights, stratification, and cluster information. The new sampling weights were constructed as the original weights divided by 7 when combining seven cycles (NHANES 2005–2020).

Values are given as weighted means, SEs for continuous variables, and frequencies for categorical variables. The Taylor series linearization method was used to calculate the variance for sub-populations of interest. The significant differences between tertiles were tested using multivariable logistic regression analysis tests for continuous variables. At the same time, we used the Rao–Scott modified chi-square tests for categorical variables, the percentages of categorical variables, and diabetes, prediabetes, and normal. We then performed an age-stratified analysis for hypertension. Furthermore, the Rao–Scott modified chi-square tests were used for the categorical variables and the percentages of categorical variables with the total MUFA intake and the total PUFA intake.

Multivariable logistic regression analysis tests for sample survey data were conducted to analyze the association between the prevalence of diabetes and UFA intake. Intake of total MUFA, PUFA, four specific fatty acids of MUFA, and seven specific fatty acids of PUFA were defined as the independent variables. Moreover, prediabetes and diabetes were the dependent variables in separate models. In the basic models, the analysis of the correlations between diabetes, prediabetes, and the intake of total MUFA, PUFA, and specific fatty acids was carried out first. In a further step, the following potential covariates affecting these associations were added: gender, age, ethnicity, high education level, poverty–income ratio, and total energy intake. Each variable was initially considered separately; only variables that had an independent significant effect on the basic models or substantially modified the principal associations between diabetes and UFA were included in the subsequent multivariable analyses. Model I was a crude model. Model II was adjusted for gender, age, ethnicity, high education level, and poverty–income ratio. Model III was additionally adjusted for total energy intake (Kcal/d).

Considering that the participants in different age groups may be a lamination factor, we further conducted multivariable logistic regression analysis tests to examine the association of total and subtypes of MUFA and PUFA intake with different age groups and diabetes, prediabetes, and normal. All statistical analyses were conducted according to the NHANES statistical tutorial (15). All of these were performed with SAS 9.4 (SAS Institute Inc., Cary, North Carolina). A two-sided *p* < 0.05 was considered statistically significant.

## Results

### Characteristics of the diabetes sample in the groups

The basic characteristics of the participants stratified by diabetes risk are presented in Table 1. A total of 16,290 participants were included in this cross-sectional analysis. In the present analysis, 52.36% of participants were female, and the median age was 47 years. From the study sample, the normal glucose tolerance, prediabetes, and T2DM percentages were 42.50, 44.34, and 13.16%, respectively. Prediabetes and diabetes were more likely to occur in older (aged ≥ 40 years) and lower educational level ( ≤ 12 years) participants (*p* < 0.001). Moreover, participants with prediabetes tended to have higher body weight and higher intakes of MUFA, PUFA, subtypes of MUFA, and subtypes of PUFA. In contrast, diabetic patients had a higher intake of PFA 18:4 and PFA 20:5 (*p* < 0.001). After stratifying according to gender, we found similar results among female participants, while in male participants, the ethnicity and poverty–income ratio were not statistically different among normal, prediabetes, and diabetes groups (Supplementary Tables S1–S4).

**Table 1:** Characteristics^a^ stratified by groups of diabetes risk sample for participants in NHANES 2005–2020.3 (*n* = 16,290).

**Characteristics**	**Normal vs. prediabetes**			**Normal vs. diabetes**		
	**Normal**	**Prediabetes^b^**	***P*-value**	**Normal**	**Diabetes^b^**	***P*-value**
*n* (%)	6,365 (47.09)	7,153 (52.91)		6,365 (69.66)	2,772 (30.34)	
Sex			< 0.001			< 0.001
Male	2,503 (39.76)	3,803 (53.59)		2,503 (39.76)	1,455 (52.76)	
Female	3,862 (60.24)	3,350 (46.41)		3,862 (60.24)	1,317 (47.24)	
Age (years)			< 0.001			< 0.001
18–39	3,643 (53.20)	1,925 (26.68)		3,643 (53.20)	229 (9.52)	
40–59	1,762 (33.59)	2,540 (40.56)		1,762 (33.59)	873 (35.71)	
≥60	960 (13.21)	2,688 (32.75)		960 (13.21)	1,670 (54.77)	
Ethnicity (*n*, %)			0.24			< 0.001
Non-Hispanic white	2,861 (69.83)	3,115 (69.37)		2,861 (69.83)	1,034 (63.58)	
Non-Hispanic black	1,268 (9.80)	1,504 (9.97)		1,268 (9.80)	739 (14.21)	
Mexican American	935 (7.54)	1,118 (8.42)		935 (7.54)	469 (9.28)	
Others	1,301 (12.83)	1,416 (12.24)		1,301 (12.83)	530 (12.93)	
Educational level (*n*, %)^c^			< 0.001			< 0.001
≤ 12 years	1,011 (11.59)	1,621 (15.23)		1,011 (11.59)	862 (21.88)	
>12 years	4,740 (88.41)	5,278 (84.77)		4,740 (88.41)	1,890 (78.12)	
missing	614 (5.00)	254 (1.85)		614 (5.00)	20 (0.38)	
Poverty–income ratio (*n*, %)^d^			0.03			< 0.001
≤ 1.30	1,822 (20.38)	1,959 (19.34)		1,822 (20.38)	815 (22.71)	
1.30–3.50	2,172 (34.83)	2,462 (36.06)		2,172 (34.83)	1,064 (41.68)	
>3.50	1,887 (44.78)	2,069 (44.60)		1,887 (44.78)	628 (35.61)	
Missing	484 (6.01)	663 (7.66)		484 (6.01)	265 (7.28)	
Body weight (kg)	76.53 ± 0.31	85.91 ± 0.39	< 0.001	76.53 ± 0.31	94.37 ± 0.63	< 0.001
Age: body weight	0.022 ± 0.00019	0.026 ± 0.00026	< 0.001	0.022 ± 0.00019	0.028 ± 0.00027	< 0.001
**Dietary variables (g)**						
Total monounsaturated fatty acids	28.27 ± 0.21	29.50 ± 0.24	< 0.001	28.27 ± 0.21	28.28 ± 0.42	< 0.001
Total polyunsaturated fatty acids	18.21 ± 0.16	18.72 ± 0.17	< 0.001	18.21 ± 0.16	18.28 ± 0.28	< 0.001
MFA 16:1 (hexadecenoic) (mg)	1128.01 ± 10.57	1192.78 ± 11.46	< 0.001	1128.01 ± 10.57	1143.86 ± 19.32	< 0.001
MFA 18:1 (octadecenoic)	26,297 ± 198.37	27,463 ± 220.78	< 0.001	26297 ± 198.37	26,348 ± 397.43	< 0.001
MFA 20:1 (eicosenoic)	282.67 ± 3.38	302.76 ± 3.48	< 0.001	282.67 ± 3.38	295.62 ± 5.05	< 0.001
MFA 22:1 (docosenoic)	29.69 ± 1.54	34.18 ± 1.34	< 0.001	29.69 ± 1.54	36.97 ± 2.09	< 0.001
PFA 18:2 (octadecadienoic)	16,106 ± 145.09	16,531 ± 148.08	< 0.001	16,106 ± 145.09	16140 ± 255.14	< 0.001
PFA 18:3 (octadecatrienoic)	1662.19 ± 18.98	1730.64 ± 22.25	< 0.001	1662.19 ± 18.98	1681.34 ± 29.44	< 0.001
PFA 18:4 (octadecatetraenoic)	12.42 ± 0.51	12.89 ± 0.52	< 0.001	12.42 ± 0.51	11.40 ± 0.60	< 0.001
PFA 20:4 (eicosatetraenoic)	138.85 ± 2.06	148.34 ± 1.74	< 0.001	138.85 ± 2.06	152.74 ± 2.57	< 0.001
PFA 20:5 (eicosapentaenoic)	34.66 ± 1.64	36.94 ± 1.56	< 0.001	34.66 ± 1.64	33.74 ± 1.91	< 0.001
PFA 22:5 (docosapentaenoic)	21.69 ± 0.50	24.08 ± 0.49	< 0.001	21.69 ± 0.50	22.67 ± 0.68	< 0.001
PFA 22:6 (docosahexaenoic)	66.85 ± 2.73	71.88 ± 2.50	< 0.001	66.85 ± 2.73	69.63 ± 3.25	< 0.001
Total energy intake (kcal/day)	2084.76 ± 11.66	2127.23 ± 13.44	< 0.001	2084.76 ± 11.66	1955.31 ± 23.89	< 0.001
Fast plasma glucose (mg/dL)	91.39 ± 0.11	105.42 ± 0.16	< 0.001	91.39 ± 0.11	157.64 ± 1.80	< 0.001
Glycohemoglobin (%)	5.19 ± 0.0051	5.55 ± 0.0072	< 0.001	5.19 ± 0.0051	7.21 ± 0.044	< 0.001
Insulin (Mu/Ml)	9.63 ± 0.19	14.26 ± 0.26	< 0.001	9.63 ± 0.19	20.57 ± 0.88	< 0.001

^a^Values are given as mean ± SE for continuous variables or frequencies for categorical variables. The significant differences between groups were tested using multivariable logistic regression analysis tests for continuous variables and Rao–Scott modified chi-square tests for categorical variables.

^b^Type 2 diabetes was defined as FPG ≥ 126 mg/Dl or HbA1c ≥ 6.5%, or self-reported use of insulin or oral agents. Prediabetes was defined as FPG 100–125 mg/Dl or HbA1c 5.7–6.5% (19).

^c^Over 12 years of school education.

^d^The ratio is the total family income divided by the poverty threshold.

Characteristics by tertiles of MUFA and PUFA are presented in Supplementary Table S5. Participants in the highest tertile of MUFA and PUFA were younger, with a higher poverty–income ratio, higher educational level, higher incidence of prediabetes, and lower rates of T2DM (*p* < 0.001).

### Association between diabetes and intake of MUFA

Odds ratios (ORs) for prediabetes and diabetes by tertiles of MUFA intake are presented in Table 2. In the present study, a higher dietary MUFA intake was associated with lower odds of prediabetes and diabetes. Participants in the highest MUFA tertile had ~50% lower odds for prediabetes and 69% lower odds for diabetes than those in the lowest tertile [prediabetes: ORs_medium_ (95% CI) = 0.72 (0.62, 0.84), ORs_highest_ (95% CI) = 0.50 (0.41, 0.60); T2DM: ORs_medium_ (95% CI) = 0.52 (0.43, 0.62), ORs_highest_ (95% CI) = 0.31 (0.24, 0.39)], after adjustment for age, gender, ethnicity, education level, family income–poverty ratio, and total energy intake.

**Table 2:** Odds ratios with 95% confidence intervals for odds for prediabetes and diabetes by total monounsaturated fatty acids and polyunsaturated fatty acids for participants in NHANES 2005–2020.3 (*n* = 16,290)^a^.

	**Normal vs. prediabetes**			**Normal vs. diabetes**		
	**Model I^b^**	**Model II^c^**	**Model III^d^**	**Model I^b^**	**Model II^c^**	**Model III^d^**
**Total monounsaturated fatty acids**						
T1	1	1	1	1	1	1
T2	0.87 (0.77, 0.98)	0.82 (0.72, 0.94)	0.72 (0.62, 0.84)	0.59 (0.51, 0.68)	0.60 (0.51, 0.70)	0.52 (0.43, 0.62)
T3	0.71 (0.64, 0.80)	0.67 (0.59, 0.77)	0.50 (0.41, 0.60)	0.40 (0.34, 0.47)	0.42 (0.35, 0.51)	0.31 (0.24, 0.39)
**Total polyunsaturated fatty acids**						
T1	1	1	1	1	1	1
T2	0.81 (0.73, 0.91)	0.81 (0.71, 0.91)	0.72 (0.64, 0.82)	0.60 (0.51, 0.71)	0.65 (0.54, 0.78)	0.58 (0.49, 0.70)
T3	0.67 (0.60, 0.74)	0.65 (0.58, 0.73)	0.51 (0.43, 0.59)	0.39 (0.33, 0.46)	0.41 (0.34, 0.50)	0.32 (0.26, 0.41)

^a^Values are odds ratio and 95% confidence interval calculated by multinomial logistic regression.

^b^Model I: crude model.

^c^Model II: adjusted for gender, age, ethnicity, educational level, and poverty–income ratio.

^d^Model III: additionally adjusted for total energy intake (Kcal/d).

**Figure 2:**
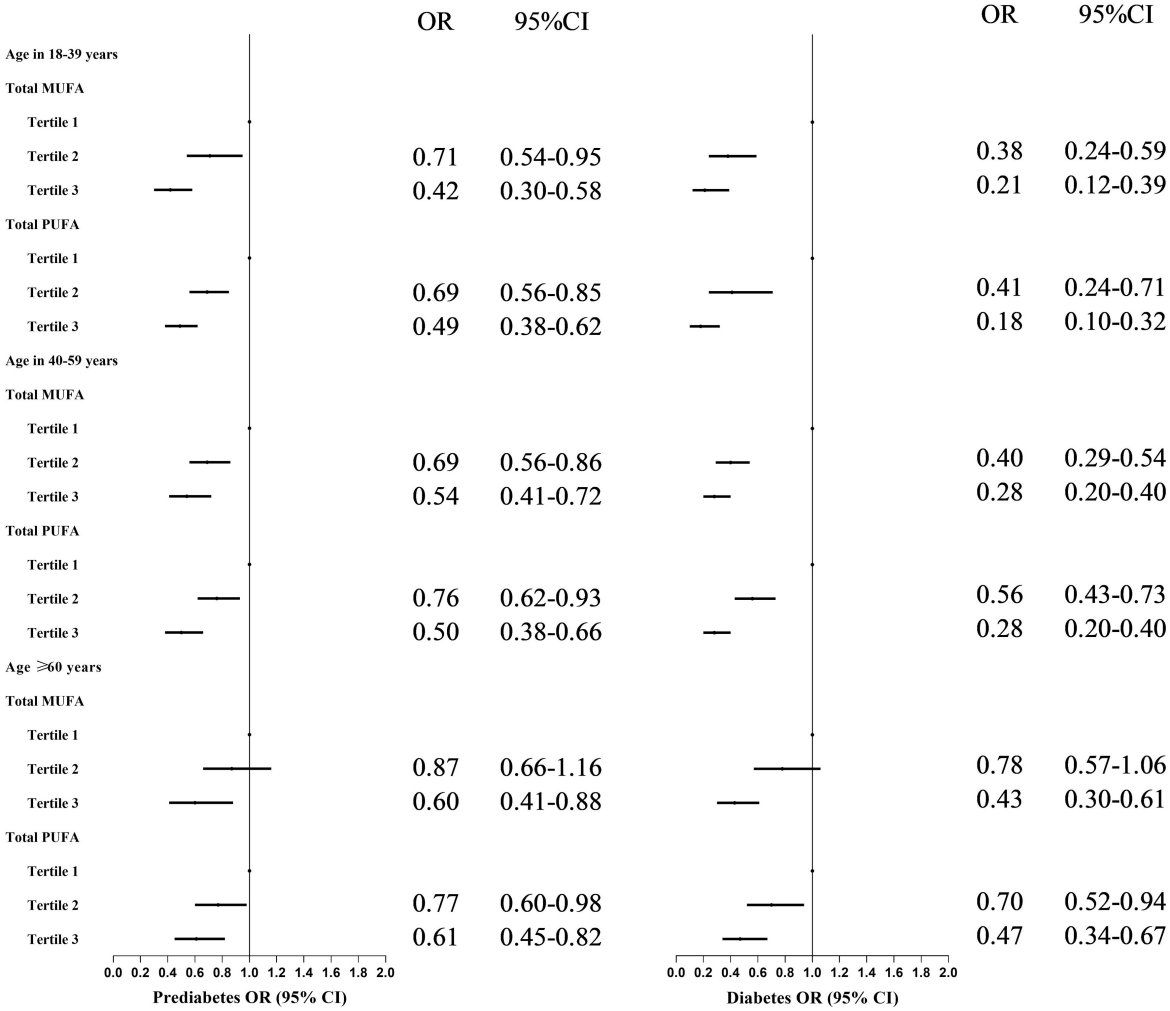
Odds ratios with 95% confidence intervals for odds for prediabetes by tertiles of total monounsaturated fatty acids and polyunsaturated fatty acids in different age groups for participants in NHANES. MUFAs, total monounsaturated fatty acids; PUFAs, total polyunsaturated fatty acids; ORs, Odds ratios; CIs, confidence intervals. OR and 95% CI were adjusted for gender, age, ethnicity, high educational level, poverty–income ratio, and total energy intake.

These identified negative associations were affected by age, weakening, and aging (Figure 2). Higher MUFA intake was inversely associated with prediabetes and T2DM risk among participants compared with the lowest MUFA intake: for participants aged 18–39, 40–59, and ≥60 years, who, in the highest MUFA tertile, had an approximately 58, 46, and 40% lower risk of prediabetes and 79, 72, and 57% lower risk of T2DM, respectively. Medium intake of MUFA was significantly associated with a lower risk of prediabetes and diabetes among participants aged 18–59 years old, not in participants aged ≥60 years.

**Figure 3:**
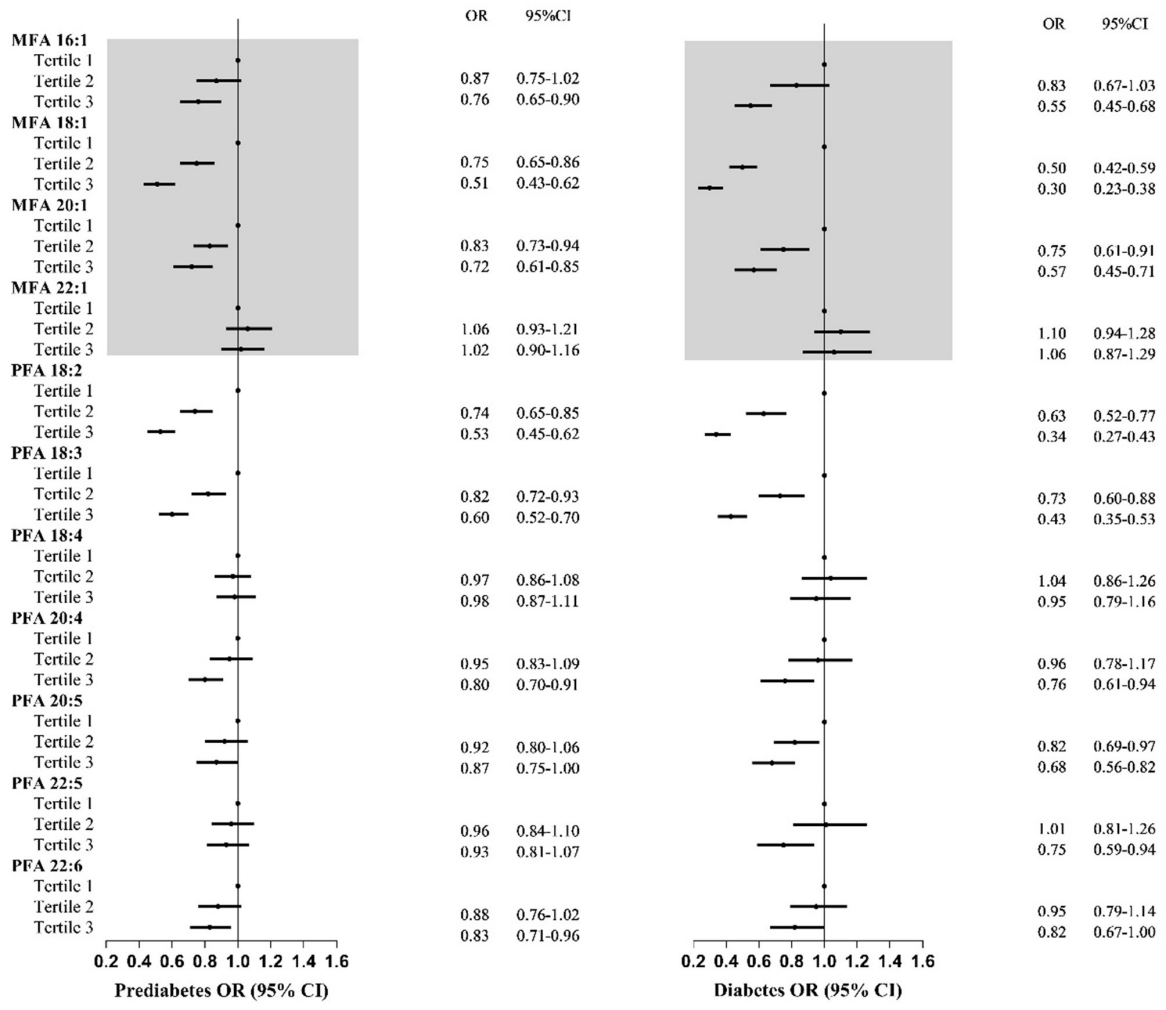
Odds ratios with 95% confidence intervals for odds for prediabetes and diabetes by tertiles of specific types of total monounsaturated fatty acids and polyunsaturated fatty acids for participants in NHANES. MUFA, total monounsaturated fatty acids; PUFAs, total polyunsaturated fatty acids; ORs, odds ratios; CIs, confidence intervals. OR and 95% CI were adjusted for gender, age, ethnicity, high educational level, poverty–income ratio, and total energy intake.

Moreover, the effects of MUFA subtypes on prediabetes and diabetes were different (Figure 3). Higher intakes of MFA 18:1 and MFA 20:1 were related to a 25–49% and 17–28% lower prediabetes risk, respectively. Participants in the highest MFA 16:1 tertile tended to have a 24% lower prediabetes risk. No association was observed between MFA 22:1 and prediabetes risk. Furthermore, similar trends were found between specific MUFAs and diabetes.

Similarly, the effects of subtypes of MUFA on prediabetes and diabetes varied among different age groups (Supplementary Figures S1, S2). MFA 16:1, MFA 18:1, and MFA 20:1 may be protective factors for prediabetes and diabetes. However, their protective effects weakened with age, from 57 to 9%. No association was observed between MFA 22:1 and prediabetes and diabetes.

### Associations between diabetes and PUFA

Like MUFA, a higher intake of PUFA was negatively associated with prediabetes and diabetes risk both in crude and adjusted models (Table 2). Participants in the higher PUFA tertile had approximately 28–49% lower odds for prediabetes and 42–68% lower odds for diabetes than those in the lowest tertile [prediabetes: ORs_medium_ (95% CI) = 0.72 (0.64, 0.82), ORs_highest_ (95% CI) = 0.51 (0.43, 0.59); diabetes: ORs_medium_ (95% CI) = 0.58 (0.49, 0.70). ORs_highest_ (95% CI) = 0.32 (0.26, 0.41)].

Similar significant results for PUFA, prediabetes, and T2DM were observed among all age groups. However, the protective effect may be weakened with age (Figure 2): participants in the highest (medium) PUFA tertile had approximately 51% (31%), 50% (24%), and 39% (23%) lower odds for prediabetes and 82% (59%), 72% (44%), and 53% (30%) for diabetes among 18–39, 40–59, and ≥60 years groups, respectively.

Adults with the highest dietary PFA 18:2 and PFA 18:3 had significantly lower prediabetes and diabetes risk (Figure 3). Higher intakes of PFA 18:2 and PFA 18:3 were negatively associated with 26–47% and 18–40% lower odds of prediabetes, with 37–66% and 27–57% of T2DM. Additionally, participants with the highest intake of PFA 20:4 and PFA 22:6 had a lower prediabetes risk. Furthermore, the highest tertile intake of PFA 20:4, PFA 20:5, and PFA 22:5 was negatively related to diabetes. No relationship between PFA 18:4 and prediabetes or diabetes was observed.

Similar age trends were observed for the association between subtypes of PUFA and prediabetes and diabetes compared with MUFA (Supplementary Figures S1, S2). Few subtypes of PUFA were associated with lower diabetes risk among older adults compared with younger adults. Among the younger age groups, almost all types of PUFA were negatively associated with prediabetes or diabetes. For older adults, only PFA 18:2 and PFA 18:3 might have protective effects on diabetes and prediabetes. Compared with that effect on prediabetes, the subtype of PUFA has a significant protective effect on diabetes.

Similar trends were observed when we conducted a correlation between specific MUFA or PUFA with hemoglobin glycation and fasting glucose (Supplementary Tables S6–S9).

## Discussion

Based on a nationally representative population sample of the United States, we found that dietary MUFA and PUFA intake and some specific MUFA/PUFA may be associated with a decreased incidence of prediabetes and T2DM. Additionally, their protective effects against T2DM might be greater than those on prediabetes. Moreover, the subgroup analysis suggested that the relevance of their MUFA, PUFA, and subtype on prediabetes and diabetes varied among different age groups, being weakened along with age. This is the first study focused on the subtypes of MUFA and PUFA fatty acid intake in prediabetes and T2DM.

A higher dietary UFA has been recognized as an essential protective factor for T2DM development by many research studies. Prevailing dietary guidelines advocated substituting saturated fatty acid (SFA) with UFA, including MUFA and PUFA, mainly based on the cardiovascular benefit (22, 23), while less is known about the effects of dietary UFA, especially MUFA, on T2DM prevention. MUFA represents a healthier alternative to saturated animal fats and has several health benefits, including preventing metabolic syndrome and its complications (24). However, the relationship between MUFA and diabetes risk is a controversial issue.

Most existing studies focused on the impact of a high-MUFA diet, not MUFA, on the metabolic factors of T2DM patients. Meta-analyses of randomized controlled trials demonstrated that consuming high-MUFA diets may reduce glycosylated hemoglobin among individuals with glucose metabolism disorders (25) and improve metabolic risk factors, including fasting plasma glucose among T2DM patients (26). Errazuriz et al. conducted a randomized controlled trial on prediabetic patients, which suggested that 12 weeks of a MUFA diet could increase hepatic fat and improve both hepatic and total insulin sensitivity (27). A prospective study of 11 offspring of obese and T2DM patients indicated that weight maintenance with a MUFA-rich diet improved homeostasis model assessment of insulin resistance and fasting pro-insulin levels in insulin-resistant subjects (28). Zhuang et al. also conducted a follow-up study and found that plasma concentrations of MUFA were positively associated with T2DM risk (29). Notably, studies of MUFA and diabetes risk are limited and controversial. A prospective nationwide cohort study from China concluded that intake of MUFA from fried plant-based foods may elevate T2DM risk among the Chinese population, while non-fried plant MUFA was not associated with the study mentioned in the reference (30). In the present analysis, we suggested that high dietary MUFA intake may be associated with a decreased incidence of prediabetes and T2DM. However, we are unaware of studies on the association between dietary intakes of subtypes of MUFA. In our analysis, not all of the subtypes of MUFA may be good for prediabetes and T2DM. Moderate and high dietary intakes of MFA 18:1 and MFA 20:1 were related to low prediabetes and diabetes risk. Large intakes, not medium, of MFA 16:1 may be a protective factor for prediabetes and T2DM risk, while dietary intake of MUFA 22:1 might not be related to diabetes risk. Overall, these findings provide a clue that the type of MUFA must first be paid close attention to when using it for impaired glucose tolerance and diabetes prevention.

Compared with MUFA, PUFA [a classification of UFA that contains two or more double bonds (31)] has been studied by most researchers (13). Many pieces of evidence from a review demonstrated that PUFA had a protective effect on T2DM development (32). Some studies showed that total PUFA intake might be associated with the increased incidence of T2DM in Europe and Australia while decreasing T2DM incidence in Asia (14). Our analysis agreed with the protective effect. A high intake of total PUFA may play a protective role for diabetes and prediabetes, especially for diabetes. Therefore, public health initiatives should be tailored to improving the total dietary PUFA intake, given the increasing trend of T2DM among Americans.

PUFA includes two series of fatty acids: *n*-3 and *n*-6 series: the former includes PFA 18:3, PFA 20:5, and PFA 22:6, whereas PFA 18:2, PFA 20:3, and PFA 20:4 are examples of the latter. The effects of *n*-3, *n*-6, and PUFA on the prevention and treatment of T2DM are also controversial, positively (10), invertedly *U*-shaped (11), negatively (12, 33), or not effectively (13), and varied among different populations (11). Additionally, epidemiology studies showed that some specific types of PUFA, such as levels of PFA 18:3 (34–36) and PFA 18:2 (14, 37), were inversely associated with a lower risk of T2DM, and PFA 22:6 may be associated with increased T2DM. PFA 18:2 and PFA 18:3 are essential fatty acids constituting 85–90% of dietary *n*-6 PUFAs in the US (38). They have been proven to improve insulin resistance and glycemia (39) and are recommended for health in most dietary guidelines (40). Being consistent with this recommendation, we suggested that higher intakes of PFA 18:2 and PFA 18:3 were beneficial for prediabetes and diabetes, especially in younger adults. However, we found no association between PFA 22:6 and PFA 20:5, also referred to as DHA and EPA, and diabetes. DHA, a major nutrient for the growth and maintenance of nervous system cells, is essential for the intellectual and visual development of the unborn baby (41). Therefore, the association between PFA 20:5, PFA 22:6, and other subtypes of PUFA should be further studied.

Nevertheless, the protective effects of MUFA or PUFA intake on diabetes risk may be related to an incretin peptide hormone, glucagon-like peptide-1 (GLP-1). GPR120 is a receptor expressed in the adipose tissue, pro-inflammatory macrophages, and gastrointestinal tract, especially in the enteroendocrine L cells (42). Notably, PUFA can bind with GPR120 to promote the release of GLP-1, which further affects insulin secretion. Moreover, this stimulatory effect has a dose-dependent relationship with the concentration of free fatty acids in the blood (43, 44).

Stratified analyses also showed a significant difference among all age groups regarding the relationship between MUFA and PUFA intake for prediabetes and T2DM. The protective effect decreased with increasing age. The reasons for this may ascribe some abnormalities among older adults in islet β-cell and insulin secretion, such as impaired insulin secretion pulsatility, decreased insulin sensitivity of pancreatic β-cells to insulinotropic gut hormones, and diminished insulin response to non-glucose stimuli that may lead to lower protective functions among older adults (45). However, it can also have protective functions when taking large amounts of specific MUFA and PUFA fatty acids. Even so, some PUFAs can still be considered an important modifiable factor for improving diabetes risk in older adults, such as PFA 18:2 and PFA 20:5.

Interestingly, we also found that the protective effects of MUFA, PUFA, or subtypes against T2DM might be greater than those of prediabetes. Prediabetes is a condition in which fast glucose is already above normal but not enough to diagnose diabetes (46). There is no doubt that diabetes is more serious than prediabetes. Our results revealed that diabetic patients may have a more severe UFA deficiency or that the therapeutic effect of UFA may be better than the preventive effect. More intervention studies are needed to confirm this.

The present analysis had the advantages of a large sample size, a strong representative of the study population from 2005 to March 2020, and comprehensive data focusing on the association between total and subtypes of MUFA and PUFA. However, there are still several limitations to this study. First, though we have a large sample size and a strong representative sample of the population from 2005 to March 2020, the NHANES was observational and did not conduct the cohort survey. Second, we collected 2 days of dietary recall interviews and took averages of these nutrients, but maybe there was memory bias. Third, the association between each subtype of MUFA and PUFA from dietary supplements and diabetes risk should be considered. Furthermore, this study did not collect the MUFA and PUFA of the supplements. Finally, the effects of potential confounding factors on the results, such as genetic susceptibility, psychosocial stress, physical activity, and body fat, still need further study.

## Conclusion

In the present analysis, we found that dietary MUFA and PUFA intake and some specific MUFA/PUFA may be associated with a decreased incidence of prediabetes and T2DM. Additionally, their protective effects against T2DM might be more significant than those with prediabetes. Moreover, subgroup analysis suggested that the relevance of their MUFA, PUFA, and subtype on prediabetes and diabetes varied among different age groups, being weakened along with age. Notably, this warrants the added investigation of the effects of the subtypes of MUFA and PUFA or their age differences on prediabetes and T2DM.

## Data availability statement

The datasets presented in this study can be found in online repositories. The names of the repository/repositories and accession number(s) can be found at: https://www.cdc.gov/nchs/nhanes/index.

## Ethics statement

The NCHS Ethics Review Board approved this study and informed consent was obtained from every participant. The studies were conducted in accordance with the local legislation and institutional requirements. The participants provided their written informed consent to participate in this study.

## Author contributions

SJ: Conceptualization, Formal analysis, Methodology, Software, Visualization, Writing – original draft. WY: Conceptualization, Validation, Visualization, Writing – original draft. YL: Data curation, Validation, Writing – review & editing. JF: Data curation, Writing – review & editing. JM: Formal analysis, Writing – review & editing. HS: Supervision, Writing – review & editing. HX: Conceptualization, Project administration, Software, Writing – original draft, Writing – review & editing.

## References

[1] World Health Organization. Global Report on Diabetes. Geneva: WHO (2016).

[2] International, Diabetes Federation (IDF). Diabetes Atlas, 10th Edn. Availabe online at: https://diabetesatlas.org/ (accessed May 26, 2023).

[3] Cho NH Shaw JE Karuranga S Huang Y da Rocha Fernandes JD Ohlrogge AW. IDFDiabetes Atlas: global estimates of diabetes prevalence for 2017 and projections for 2045. Diabetes Res Clin Pract. (2018) 138:271–81. 10.1016/j.diabres.2018.02.023 29496507

[4] Palacios OM Kramer M Maki KC. Diet and prevention of type 2 diabetes mellitus: beyond weight loss and exercise. Expert Rev Endocrinol Metab. (2019) 14:1–12. 10.1080/17446651.2019.1554430 30521416

[5] González-Becerra K Ramos-Lopez O Barrón-Cabrera E Riezu-Boj JI Milagro FI Martínez-López E. Fatty acids, epigenetic mechanisms and chronic diseases: a systematic review. Lipids Health Dis. (2019) 18:178. 10.1186/s12944-019-1120-6 31615571 PMC6792183

[6] Wolf G. Role of fatty acids in the development of insulin resistance and type 2 diabetes mellitus. Nutr Rev. (2008) 66:597–600. 10.1111/j.1753-4887.2008.00110.x 18826455

[7] Bozzetto L Prinster A Annuzzi G Costagliola L Mangione A Vitelli A. Liver fat is reduced by an isoenergetic MUFA diet in a controlled randomized study in type 2 diabetic patients. Diabetes Care. (2012) 35:1429–35. 10.2337/dc12-0033 22723581 PMC3379578

[8] Shapiro H Theilla M Attal-Singer J Singer P. Effects of polyunsaturated fatty acid consumption in diabetic nephropathy. Nat Rev Nephrol. (2011) 7:110–21. 10.1038/nrneph.2010.156 21135888

[9] Martín-Peláez S Fito M Castaner O. Mediterranean diet effects on type 2 diabetes prevention, disease progression, and related mechanisms. A review. Nutrients. (2020) 12:2236. 10.3390/nu12082236 32726990 PMC7468821

[10] Zhou Y Tian C Jia C. Association of fish and n-3 fatty acid intake with the risk of type 2 diabetes: a meta-analysis of prospective studies. Br J Nutr. (2012) 108:408–17. 10.1017/S0007114512002036 22857650

[11] Chen C Yang Y Yu X Hu S Shao S. Association between omega-3 fatty acids consumption and the risk of type 2 diabetes: a meta-analysis of cohort studies. J Diabetes Investig. (2017) 8:480–8. 10.1111/jdi.12614 28032469 PMC5497038

[12] Qian F Ardisson Korat AV Imamura F Marklund M Tintle N Virtanen JK. n-3 fatty acid biomarkers and incident type 2 diabetes: an individual participant-level pooling project of 20 prospective cohort studies. Diabetes Care. (2021) 44:1133–42. 10.2337/dc20-2426 33658295 PMC8132316

[13] Brown TJ Brainard J Song F Wang X Abdelhamid A Hooper L. Omega-3, omega-6, and total dietary polyunsaturated fat for prevention and treatment of type 2 diabetes mellitus: systematic review and meta-analysis of randomised controlled trials. BMJ. (2019) 366:l4697. 10.1136/bmj.l4697 31434641 PMC6699594

[14] Hu M Fang Z Zhang T Chen Y. Polyunsaturated fatty acid intake and incidence of type 2 diabetes in adults: a dose response meta-analysis of cohort studies. Diabetol Metab Syndr. (2022) 14:34. 10.1186/s13098-022-00804-1 35241134 PMC8892771

[15] National Center for Health Statistics National Health and Nutrition Examination Survey. Available online at: https://www.cdc.gov/nchs/nhanes/index.htm (accessed January 10, 2023).

[16] Zipf G Chiappa M Porter KS Ostchega Y Lewis BG Dostal J. National health and nutrition examination survey: plan and operations, 1999-2010. Vital Health Stat. (2013) 1:1–37. 25078429

[17] NHANES- NCHS Research Ethics Review Board Approval. Availabe online at: https://www.cdc.gov/nchs/nhanes/irba98.htm (accessed on May 28, 2023).

[18] Park SW Kim DY Bak GT Hyun DS Kim SK. Relation of dietary n-3 and n-6 fatty acid intakes to metabolic syndrome in middle-aged people depending on the level of HbA1c: a review of national health and nutrition survey data from 2014 to 2016. Medicina. (2022) 58:1017. 10.3390/medicina58081017 36013484 PMC9413490

[19] National Health and Nutrition Examination Survey 2017-March 2020 Data Documentation Codebook and Frequencies. Availabe online at: https://wwwn.cdc.gov/Nchs/Nhanes/2017-2018/P_FASTQX.htm#Eligible_Sample (accessed January 10, 2023).

[20] Gloyn AL Drucker DJ. Precision medicine in the management of type 2 diabetes. Lancet Diabetes Endocrinol. (2018) 6:891–900. 10.1016/S2213-8587(18)30052-4 29699867

[21] Ahluwalia N Dwyer J Terry A Moshfegh A Johnson C. Update on NHANES dietary data: focus on collection, release, analytical considerations, and uses to inform public policy. Adv Nutr. (2016) 7:121–34. 10.3945/an.115.009258 26773020 PMC4717880

[22] Hooper L Martin N Jimoh OF Kirk C Foster E Abdelhamid AS. Reduction in saturated fat intake for cardiovascular disease. Cochrane Database Syst Rev. (2020) 5:Cd011737. 10.1002/14651858.CD011737.pub2 32428300 PMC7388853

[23] Schwingshackl L Hoffmann G. Monounsaturated fatty acids and risk of cardiovascular disease: synopsis of the evidence available from systematic reviews and meta-analyses. Nutrients. (2012) 4:1989–2007. 10.3390/nu4121989 23363996 PMC3546618

[24] Schwingshackl L Strasser B Hoffmann G. Effects of monounsaturated fatty acids on glycaemic control in patients with abnormal glucose metabolism: a systematic review and meta-analysis. Ann Nutr Metab. (2011) 58:290–6. 10.1159/000331214 21912106

[25] Qian F Korat AA Malik V Hu FB. Metabolic effects of monounsaturated fatty acid-enriched diets compared with carbohydrate or polyunsaturated fatty acid-enriched diets in patients with type 2 diabetes: a systematic review and meta-analysis of randomized controlled trials. Diabetes Care. (2016) 39:1448–57. 10.2337/dc16-0513 27457635 PMC4955926

[26] Schwingshackl L Strasser B. High-MUFA diets reduce fasting glucose in patients with type 2 diabetes. Ann Nutr Metab. (2012) 60:33–4. 10.1159/000335162 22212514

[27] Errazuriz I Dube S Slama M Visentin R Nayar S O'Connor H. Randomized controlled trial of a MUFA or fiber-rich diet on hepatic fat in prediabetes. J Clin Endocrinol Metab. (2017) 102:1765–74. 10.1210/jc.2016-3722 28323952 PMC5443322

[28] Paniagua JA de la Sacristana AG Sánchez E Romero I Vidal-Puig A Berral FJ. A MUFA-rich diet improves posprandial glucose, lipid and GLP-1 responses in insulin-resistant subjects. J Am Coll Nutr. (2007) 26:434–44. 10.1080/07315724.2007.10719633 17914131

[29] Zhuang P Liu X Li Y Li H Zhang L Wan X. Circulating fatty acids and genetic predisposition to type 2 diabetes: gene-nutrient interaction analysis. Diabetes Care. (2022) 45:564–75. 10.2337/dc21-2048 35089324

[30] Zhuang P Zhang Y Mao L Wang L Wu F Cheng L. The association between consumption of monounsaturated fats from animal- vs. plant-based foods and the risk of type 2 diabetes: a prospective nationwide cohort study. Br J Nutr. (2020) 124:102–11. 10.1017/S0007114520000677 32102700

[31] Das UN. Polyunsaturated fatty acids and sepsis. Nutrition. (2019) 65:39–43. 10.1016/j.nut.2019.02.016 31029920

[32] Carrasquilla GD Jakupović H Kilpeläinen TO. Dietary fat and the genetic risk of type 2 diabetes. Curr Diab Rep. (2019) 19:109. 10.1007/s11892-019-1251-1 31686257

[33] Xiao Y Zhang Q Liao X Elbelt U Weylandt KH. The effects of omega-3 fatty acids in type 2 diabetes: A systematic review and meta-analysis. Prostaglandins Leukot Essent Fatty Acids. (2022) 182:102456. 10.1016/j.plefa.2022.102456 35717726

[34] Jiang H Wang L Wang D Yan N Li C Wu M. Omega-3 polyunsaturated fatty acid biomarkers and risk of type 2 diabetes, cardiovascular disease, cancer, and mortality. Clin Nutr. (2022) 41:1798–807. 10.1016/j.clnu.2022.06.034 35830775

[35] Xu B Xu Z Xu D Tan X. Effect of n-3 polyunsaturated fatty acids on ischemic heart disease and cardiometabolic risk factors: a two-sample Mendelian randomization study. BMC Cardiovasc Disord. (2021) 21:532. 10.1186/s12872-021-02342-6 34749668 PMC8576934

[36] Wu J HY Marklund M Imamura F Tintle N Ardisson Korat AV de Goede J. Omega-6 fatty acid biomarkers and incident type 2 diabetes: pooled analysis of individual-level data for 39 740 adults from 20 prospective cohort studies. Lancet Diabetes Endocrinol. (2017) 5:965–74. 10.1016/S2213-8587(17)30307-8 29032079 PMC6029721

[37] Mousavi SM Jalilpiran Y Karimi E Aune D Larijani B Mozaffarian D. Dietary intake of linoleic acid, its concentrations, and the risk of type 2 diabetes: a systematic review and dose-response meta-analysis of prospective cohort studies. Diabetes Care. (2021) 44:2173–81. 10.2337/dc21-0438 34417277

[38] Russo GL. Dietary n-6 and n-3 polyunsaturated fatty acids: from biochemistry to clinical implications in cardiovascular prevention. Biochem Pharmacol. (2009) 77:937–46. 10.1016/j.bcp.2008.10.020 19022225

[39] Imamura F Micha R Wu JH de Oliveira Otto MC Otite FO Abioye AI. Effects of saturated fat, polyunsaturated fat, monounsaturated fat, and carbohydrate on glucose-insulin homeostasis: a systematic review and meta-analysis of randomised controlled feeding trials. PLoS Med. (2016) 13:e1002087. 10.1371/journal.pmed.1002087 27434027 PMC4951141

[40] McGuire S. Scientific Report of the 2015 Dietary Guidelines Advisory Committee. Washington, DC. US Departments of Agriculture and Health and Human Services, 2015. Adv Nutr. (2016) 7:202–4. 10.3945/an.115.011684 26773024 PMC4717899

[41] Horrocks LA Yeo YK. Health benefits of docosahexaenoic acid (DHA). Pharmacol Res. (1999) 40:211–25. 10.1006/phrs.1999.0495 10479465

[42] Song T Yang Y Zhou Y Wei H Peng J. GPR120: a critical role in adipogenesis, inflammation, and energy metabolism in adipose tissue. Cell Mol Life Sci. (2017) 74:2723–33. 10.1007/s00018-017-2492-2 28285320 PMC11107682

[43] Hirasawa A Tsumaya K Awaji T Katsuma S Adachi T Yamada M. Free fatty acids regulate gut incretin glucagon-like peptide-1 secretion through GPR120. Nat Med. (2005) 11:90–4. 10.1038/nm1168 15619630

[44] Oh DY Talukdar S Bae EJ Imamura T Morinaga H Fan W. GPR120 is an omega-3 fatty acid receptor mediating potent anti-inflammatory and insulin-sensitizing effects. Cell. (2010) 142:687–98. 10.1016/j.cell.2010.07.041 20813258 PMC2956412

[45] Scheen AJ. Diabetes mellitus in the elderly: insulin resistance and/or impaired insulin secretion? Diabetes Metab. (2005) 2:5S27–34. 10.1016/S1262-3636(05)73649-1 16415763

[46] Tabák AG Herder C Rathmann W Brunner EJ Kivimäki M. Prediabetes: a high-risk state for diabetes development. Lancet. (2012) 379:2279–90. 10.1016/S0140-6736(12)60283-9 22683128 PMC3891203

