# Supplementary material for: Monounsaturated and polyunsaturated fatty acids concerning prediabetes and type 2 diabetes mellitus risk among participants in the National Health and Nutrition Examination Surveys (NHANES) from 2005 to March 2020
Source: Front Nutr. 2023 Nov 24;10:1284800. doi: 10.3389/fnut.2023.1284800 (PMC10704138; doi:10.3389/fnut.2023.1284800)
Supplement: Supplementary file 1 [file Data_Sheet_1.docx]

**Table S1**. Characteristics^1^ stratified by groups of diabetes risk sample for participants in NHANES 2005-2020.3(n = 16,290).

| **Characteristics** | **Diabetes Risk Sample^2^** | | | ***p* value** |
| --- | --- | --- | --- | --- |
|  | **Normal** | **Prediabetes** | **Diabetes** |  |
| **Male** |  |  |  |  |
| **n (%)** | 2503 (32.25) | 3803 (49.00) | 1455 (18.75) |  |
| **Age (years)** |  |  |  | <0.001 |
| 18-39 | 1445(55.14) | 1147(31.47) | 118(8.99) |  |
| 40-59 | 659(32.83) | 1320(40.83) | 455(37.48) |  |
| ≥60 | 399(12.04) | 1336(27.70) | 882(53.54) |  |
| **Ethnicity (n, %)** |  |  |  | 0.055 |
| Non-Hispanic white | 1139(70.34) | 1711(69.95) | 593(66.73) |  |
| Non-Hispanic black | 521(9.59) | 753(8.54) | 341(11.47) |  |
| Mexican American | 362(8.01) | 585(9.01) | 241(9.49) |  |
| Others | 481(12.06) | 754(12.49) | 280(12.31) |  |
| **Educational level (n, %)**^3^ |  |  |  | <0.001 |
| ≤12 years | 451(12.70) | 862(15.26) | 429(19.37) |  |
| ＞12 years | 1759(81.04) | 2775(82.33) | 1015(80.14) |  |
| missing | 293(6.26) | 166(2.41) | 11(0.48) |  |
| **Poverty-income ratio (n, %)**^4^ |  |  |  | 0.19 |
| ≤1.30 | 672(17.77) | 971(16.11) | 367(17.45) |  |
| 1.30-3.50 | 865(33.58) | 1293(31.94) | 564(36.07) |  |
| >3.50 | 766(42.35) | 1199(44.82) | 394(39.41) |  |
| missing | 200(6.30) | 340(7.13) | 130(7.07) |  |
| **Body weight (kg)**  **Age: body weight** | 84.47±0.51  0.019±0.00025 | 90.77±0.45  0.023±0.00025 | 98.65±1.03  0.026±0.00041 | <0.001  <0.001 |
| **Dietary variables (g)** |  |  |  |  |
| Total monounsaturated fatty acids | 33.63±0.39 | 34.00±0.31 | 32.16±0.57 | <0.001 |
| Total polyunsaturated fatty acids | 20.91±0.27 | 20.92±0.25 | 20.27±0.40 | <0.001 |
| MFA 16:1 (Hexadecenoic)(mg) | 1403.00±21.80 | 1398.27±14.62 | 1323.35±27.16 | <0.001 |
| MFA 18:1 (Octadecenoic) | 31200.60±368.74 | 31604.82±294.94 | 29937.31±531.79 | <0.001 |
| MFA 20:1 (Eicosenoic) | 338.56±5.86 | 354.22±5.12 | 343.14±8.04 | <0.001 |
| MFA 22:1 (Docosenoic) | 38.83±3.51 | 42.09±2.00 | 45.99±2.98 | <0.001 |
| PFA 18:2 (Octadecadienoic) | 18512.78±242.58 | 18488.50±221.82 | 17911.33±356.08 | <0.001 |
| PFA 18:3 (Octadecatrienoic) | 1851.74±28.49 | 1902.21±34.25 | 1842.60±41.25 | <0.001 |
| PFA 18:4 (Octadecatetraenoic) | 15.40±0.87 | 15.91±0.75 | 12.88±0.96 | <0.001 |
| PFA 20:4 (Eicosatetraenoic) | 171.77±3.73 | 172.82±2.35 | 173.76±3.62 | <0.001 |
| PFA 20:5 (Eicosapentaenoic) | 38.19±2.69 | 42.72±2.19 | 38.45±2.92 | <0.001 |
| PFA 22:5 (Docosapentaenoic) | 25.90±0.89 | 27.85±0.65 | 26.37±1.00 | <0.001 |
| PFA 22:6 (Docosahexaenoic) | 75.38±4.70 | 80.88±3.61 | 80.56±5.04 | <0.001 |
| Total energy intake (kcal/day) | 2482.79±21.13 | 2437.82±17.56 | 2209.19±32.96 | <0.001 |
| **Fast plasma glucose (mg/dL)** | 92.49±0.16 | 106.11±0.17 | 161.89±2.54 | <0.001 |
| **Hemoglobin A1c (%)** | 5.18±0.0076 | 5.49±0.0090 | 7.24±0.067 | <0.001 |
| **Insulin (Μu/Ml)** | 10.26±0.32 | 14.17±0.35 | 21.02±1.52 | <0.001 |
| **Female** |  |  |  |  |
| **n (%)** | 3862 (45.28) | 3350 (39.28) | 1317 (15.44) |  |
| **Age (years)** |  |  |  | <0.001 |
| 18-39 | 2198 (51.93) | 778 (21.16) | 111 (10.11) |  |
| 40-59 | 1103 (34.09) | 1220 (40.25) | 418 (33.74) |  |
| ≥60 | 561 (13.98) | 1352 (38.59) | 788 (56.15) |  |
| **Ethnicity (n, %)** |  |  |  | <0.001 |
| Non-Hispanic white | 1722(69.50) | 1404(68.70) | 441(60.06) |  |
| Non-Hispanic black | 747(9.94) | 751(11.61) | 398(17.28) |  |
| Mexican American | 573(7.23) | 533(7.74) | 228(9.04) |  |
| Others | 820(13.34) | 662(11.95) | 250(13.62) |  |
| **Educational level (n, %)**^3^ |  |  |  | <0.001 |
| ≤12 years | 560(9.89) | 759(14.60) | 433(24.49) |  |
| ＞12 years | 2981(85.95) | 2503(84.20) | 875(75.24) |  |
| missing | 321(4.17) | 88(1.20) | 9(0.27) |  |
| **Poverty-income ratio (n, %)**^4^ |  |  |  | <0.001 |
| ≤1.30 | 1150(20.07) | 988(19.88) | 448(25.09) |  |
| 1.30-3.50 | 1307(32.18) | 1169(34.85) | 500(41.52) |  |
| >3.50 | 1121(41.93) | 870(37.00) | 234(25.87) |  |
| missing | 284(5.82) | 323(8.28) | 135(7.52) |  |
| **Body weight (kg)**  **Age: body weight** | 71.28±0.40  0.024±0.00026 | 80.30±0.59  0.029±0.00038 | 89.59±0.89  0.030±0.00044 | <0.001  <0.001 |
| **Dietary variables (g)** |  |  |  |  |
| Total monounsaturated fatty acids | 24.73±0.21 | 24.31±0.29 | 23.95±0.58 | <0.001 |
| Total polyunsaturated fatty acids | 16.44±0.16 | 16.18±0.19 | 16.05±0.40 | <0.001 |
| MFA 16:1 (Hexadecenoic)(mg) | 946.52±9.19 | 955.49±14.21 | 943.42±22.76 | <0.001 |
| MFA 18:1 (Octadecenoic) | 23060.06±195.92 | 22680.29±271.58 | 22339.95±550.58 | <0.001 |
| MFA 20:1 (Eicosenoic) | 245.79±3.64 | 243.33±4.53 | 242.56±6.36 | <0.001 |
| MFA 22:1 (Docosenoic) | 23.65±0.92 | 25.04±1.48 | 26.89±2.69 | <0.001 |
| PFA 18:2 (Octadecadienoic) | 14517.69±142.70 | 14269.81±169.43 | 14162.90±357.13 | <0.001 |
| PFA 18:3 (Octadecatrienoic) | 1537.09±23.81 | 1532.53±22.13 | 1501.24±40.02 | <0.001 |
| PFA 18:4 (Octadecatetraenoic) | 10.46±0.52 | 9.40±0.60 | 9.75±0.81 | <0.001 |
| PFA 20:4 (Eicosatetraenoic) | 117.13±1.84 | 120.07±2.02 | 129.26±3.89 | <0.001 |
| PFA 20:5 (Eicosapentaenoic) | 32.34±1.69 | 30.26±1.79 | 28.48±2.53 | <0.001 |
| PFA 22:5 (Docosapentaenoic) | 18.92±0.50 | 19.74±0.64 | 18.55±0.80 | <0.001 |
| PFA 22:6 (Docosahexaenoic) | 61.22±2.60 | 61.49±2.85 | 57.43±3.86 | <0.001 |
| Total energy intake (kcal/day) | 1822.06±11.60 | 1768.57±14.29 | 1671.79±28.62 | <0.001 |
| **Fast plasma glucose (mg/dL)** | 90.62±0.14 | 104.48±0.20 | 153.40±1.94 | <0.001 |
| **Hemoglobin A1c (%)** | 5.19±0.0060 | 5.62±0.0079 | 7.18±0.055 | <0.001 |
| **Insulin (Μu/Ml)** | 9.20±0.21 | 14.38±0.33 | 20.08±0.73 | <0.001 |

**Table S2**. Characteristics^1^ stratified by groups of diabetes risk sample for participants in NHANES 2005-2020.3(n= 16,290).

| **Characteristics** | **Diabetes Risk Sample^2^** | | | **p value** |
| --- | --- | --- | --- | --- |
|  | **Normal** | **Prediabetes** | **Diabetes** |  |
| **n (%)** | 6365 (42.50) | 7153 (44.34) | 2772 (13.16) |  |
| **Sex** |  |  |  | <0.001 |
| Male (n, %) | 2503 (39.76) | 3803 (53.59) | 1455 (52.76) |  |
| Female | 3862 (60.24) | 3350 (46.41) | 1317 (47.24) |  |
| **Age (years)** |  |  |  | <0.001 |
| 18-39 | 3643 (53.20) | 1925 (26.68) | 229 (9.52) |  |
| 40-59 | 1762 (33.59) | 2540 (40.56) | 873 (35.71) |  |
| ≥60 | 960 (13.21) | 2688 (32.75) | 1670 (54.77) |  |
| **Ethnicity (n, %)** |  |  |  | <0.001 |
| Non-Hispanic white | 2861 (69.83) | 3115 (69.37) | 1034 (63.58) |  |
| Non-Hispanic black | 1268 (9.80) | 1504 (9.97) | 739 (14.21) |  |
| Mexican American | 935 (7.54) | 1118 (8.42) | 469 (9.28) |  |
| Others | 1301 (12.83) | 1416 (12.24) | 530 (12.93) |  |
| **Educational level (n, %)** |  |  |  | <0.001 |
| ≤12 years | 1011 (11.59) | 1621 (15.23) | 862 (21.88) |  |
| ＞12 years | 4740 (88.41) | 5278 (84.77) | 1890 (78.12) |  |
| **Poverty-income ratio (n, %)**^3^ |  |  |  | <0.001 |
| ≤1.30 | 1822 (20.38) | 1959 (19.34) | 815 (22.71) |  |
| 1.30-3.50 | 2172 (34.83) | 2462 (36.06) | 1064 (41.68) |  |
| >3.50 | 1887 (44.78) | 2069 (44.60) | 628 (35.61) |  |
| **Body weight (kg)** | 76.53±0.31 | 85.91±0.39 | 94.37±0.63 | <0.001 |
| **Age: body weight** | 0.022±0.00019 | 0.026±0.00026 | 0.028±0.00027 | <0.001 |
| **Dietary variables (g)** |  |  |  |  |
| Total monounsaturated fatty acids | 28.27±0.21 | 29.50±0.24 | 28.28±0.42 | <0.001 |
| Total polyunsaturated fatty acids | 18.21±0.16 | 18.72±0.17 | 18.28±0.28 | <0.001 |
| MFA 16:1 (Hexadecenoic)(mg) | 1128.01±10.57 | 1192.78±11.46 | 1143.86±19.32 | <0.001 |
| MFA 18:1 (Octadecenoic) | 26297±198.37 | 27463±220.78 | 26348±397.43 | <0.001 |
| MFA 20:1 (Eicosenoic) | 282.67±3.38 | 302.76±3.48 | 295.62±5.05 | <0.001 |
| MFA 22:1 (Docosenoic) | 29.69±1.54 | 34.18±1.34 | 36.97±2.09 | <0.001 |
| PFA 18:2 (Octadecadienoic) | 16106±145.09 | 16531±148.08 | 16140±255.14 | <0.001 |
| PFA 18:3 (Octadecatrienoic) | 1662.19±18.98 | 1730.64±22.25 | 1681.34±29.44 | <0.001 |
| PFA 18:4 (Octadecatetraenoic) | 12.42±0.51 | 12.89±0.52 | 11.40±0.60 | <0.001 |
| PFA 20:4 (Eicosatetraenoic) | 138.85±2.06 | 148.34±1.74 | 152.74±2.57 | <0.001 |
| PFA 20:5 (Eicosapentaenoic) | 34.66±1.64 | 36.94±1.56 | 33.74±1.91 | <0.001 |
| PFA 22:5 (Docosapentaenoic) | 21.69±0.50 | 24.08±0.49 | 22.67±0.68 | <0.001 |
| PFA 22:6 (Docosahexaenoic) | 66.85±2.73 | 71.88±2.50 | 69.63±3.25 | <0.001 |
| **Total energy intake (Kcal/day)** | 2084.76±11.66 | 2127.23±13.44 | 1955.31±23.89 | <0.001 |
| **Fast plasma glucose (mg/dL)** | 91.39±0.11 | 105.42±0.16 | 157.64±1.80 | <0.001 |
| **Hemoglobin A1c (%)** | 5.19±0.0051 | 5.55±0.0072 | 7.21±0.044 | <0.001 |
| **Insulin (Μu/Ml)** | 9.63±0.19 | 14.26±0.26 | 20.57±0.88 | <0.001 |

^1^ Values are given as mean±SE for continuous variables, or frequencies for categorical variables. The significant differences between groups were tested using Multivariable logistic regression analyses tests for continuous variables, and Rao-Scott modified chi-square tests for categorical variables.

^2^ Type 2 diabetes was defined as FPG≥126 mg/Dl or HbA1c≥6.5%, or self-reported use of insulin or oral agents. Prediabetes was defined as FPG 100-125 mg/Dl or HbA1c 5.7%-6.5% (22)**.**

**Table S3**. Characteristics^1^ stratified by groups of diabetes risk sample for males in NHANES 2005-2020.3(n = 7,761).

| **Characteristics** | **Normal vs. Prediabetes** | | |  | **Normal vs. Diabetes** | | |
| --- | --- | --- | --- | --- | --- | --- | --- |
|  | **Normal** | **Prediabetes** | ***P* value** |  | **Normal** | **Diabetes** | ***P* value** |
| **n (%)** | 2503 (39.69) | 3803 (60.31) |  |  | 2503 (63.24) | 1455 (36.76) |  |
| **Age (years)** |  |  | <0.001 |  |  |  | <0.001 |
| 18-39 | 1445(55.14) | 1147(31.47) |  |  | 1445(55.14) | 118(8.99) |  |
| 40-59 | 659(32.83) | 1320(40.83) |  |  | 659(32.83) | 455(37.48) |  |
| ≥60 | 399(12.04) | 1336(27.70) |  |  | 399(12.04) | 882(53.54) |  |
| **Ethnicity (n, %)** |  |  | 0.33 |  |  |  | 0.09 |
| Non-Hispanic white | 1139(70.34) | 1711(69.95) |  |  | 1139(70.34) | 593(66.73) |  |
| Non-Hispanic black | 521(9.59) | 753(8.54) |  |  | 521(9.59) | 341(11.47) |  |
| Mexican American | 362(8.01) | 585(9.01) |  |  | 362(8.01) | 241(9.49) |  |
| Others | 481(12.06) | 754(12.49) |  |  | 481(12.06) | 280(12.31) |  |
| **Educational level (n, %)**^3^ |  |  | <0.001 |  |  |  | <0.001 |
| ≤12 years | 451(12.70) | 862(15.26) |  |  | 451(12.70) | 429(19.37) |  |
| ＞12 years | 1759(81.04) | 2775(82.33) |  |  | 1759(81.04) | 1015(80.14) |  |
| missing | 293(6.26) | 166(2.41) |  |  | 293(6.26) | 11(0.48) |  |
| **Poverty-income ratio (n, %)**^4^ |  |  | 0.31 |  |  |  | 0.51 |
| ≤1.30 | 672(17.77) | 971(16.11) |  |  | 672(17.77) | 367(17.45) |  |
| 1.30-3.50 | 865(33.58) | 1293(31.94) |  |  | 865(33.58) | 564(36.07) |  |
| >3.50 | 766(42.35) | 1199(44.82) |  |  | 766(42.35) | 394(39.41) |  |
| missing | 200(6.30) | 340(7.13) |  |  | 200(6.30) | 130(7.07) |  |
| **Body weight (kg)**  **Age: body weight** | 84.47±0.51  0.019±0.00025 | 90.77±0.45  0.023±0.00025 | <0.001  <0.001 |  | 84.47±0.51  0.019±0.00025 | 98.65±1.03  0.026±0.00041 | <0.001  <0.001 |
| **Dietary variables (g)** |  |  |  |  |  |  |  |
| Total monounsaturated fatty acids | 33.63±0.39 | 34.00±0.31 | <0.001 |  | 33.63±0.39 | 32.16±0.57 | <0.001 |
| Total polyunsaturated fatty acids | 20.91±0.27 | 20.92±0.25 | <0.001 |  | 20.91±0.27 | 20.27±0.40 | <0.001 |
| MFA 16:1 (Hexadecenoic)(mg) | 1403.00±21.80 | 1398.27±14.62 | <0.001 |  | 1403.00±21.80 | 1323.35±27.16 | <0.001 |
| MFA 18:1 (Octadecenoic) | 31200.60±368.74 | 31604.82±294.94 | <0.001 |  | 31200.60±368.74 | 29937.31±531.79 | <0.001 |
| MFA 20:1 (Eicosenoic) | 338.56±5.86 | 354.22±5.12 | <0.001 |  | 338.56±5.86 | 343.14±8.04 | <0.001 |
| MFA 22:1 (Docosenoic) | 38.83±3.51 | 42.09±2.00 | <0.001 |  | 38.83±3.51 | 45.99±2.98 | <0.001 |
| PFA 18:2 (Octadecadienoic) | 18512.78±242.58 | 18488.50±221.82 | <0.001 |  | 18512.78±242.58 | 17911.33±356.08 | <0.001 |
| PFA 18:3 (Octadecatrienoic) | 1851.74±28.49 | 1902.21±34.25 | <0.001 |  | 1851.74±28.49 | 1842.60±41.25 | <0.001 |
| PFA 18:4 (Octadecatetraenoic) | 15.40±0.87 | 15.91±0.75 | <0.001 |  | 15.40±0.87 | 12.88±0.96 | <0.001 |
| PFA 20:4 (Eicosatetraenoic) | 171.77±3.73 | 172.82±2.35 | <0.001 |  | 171.77±3.73 | 173.76±3.62 | <0.001 |
| PFA 20:5 (Eicosapentaenoic) | 38.19±2.69 | 42.72±2.19 | <0.001 |  | 38.19±2.69 | 38.45±2.92 | <0.001 |
| PFA 22:5 (Docosapentaenoic) | 25.90±0.89 | 27.85±0.65 | <0.001 |  | 25.90±0.89 | 26.37±1.00 | <0.001 |
| PFA 22:6 (Docosahexaenoic) | 75.38±4.70 | 80.88±3.61 | <0.001 |  | 75.38±4.70 | 80.56±5.04 | <0.001 |
| Total energy intake (kcal/day) | 2482.79±21.13 | 2437.82±17.56 | <0.001 |  | 2482.79±21.13 | 2209.19±32.96 | <0.001 |
| **Fast plasma glucose (mg/dL)** | 92.49±0.16 | 106.11±0.17 | <0.001 |  | 92.49±0.16 | 161.89±2.54 | <0.001 |
| **Hemoglobin A1c (%)** | 5.18±0.0076 | 5.49±0.0090 | <0.001 |  | 5.18±0.0076 | 7.24±0.067 | <0.001 |
| **Insulin (Μu/Ml)** | 10.26±0.32 | 14.17±0.35 | <0.001 |  | 10.26±0.32 | 21.02±1.52 | <0.001 |

^1^ Values are given as mean±SE for continuous variables, or frequencies for categorical variables. The significant differences between groups were tested using Multivariable logistic regression analyses tests for continuous variables, and Rao-Scott modified chi-square tests for categorical variables.

^2^ Type 2 diabetes was defined as FPG≥126 mg/Dl or HbA1c≥6.5%, or self-reported use of insulin or oral agents. Prediabetes was defined as FPG 100-125 mg/Dl or HbA1c 5.7%-6.5% **(22)**

^3^ Over 12 years of school education.

^4^ The ratio is the total family income divided by the poverty threshold.

**Table S4**. Characteristics^1^ stratified by groups of diabetes risk sample for females in NHANES 2005-2020.3(n = 8,529).

| **Characteristics** | **Normal vs. Prediabetes** | | |  | **Normal vs. Diabetes** | | |
| --- | --- | --- | --- | --- | --- | --- | --- |
|  | **Normal** | **Prediabetes** | ***P* value** |  | **Normal** | **Diabetes** | ***P* value** |
| **n (%)** | 3862(53.55) | 3350(46.45) |  |  | 3862(74.57) | 1317(25.43) |  |
| **Age (years)** |  |  | <0.001 |  |  |  | <0.001 |
| 18-39 | 2198(51.93) | 778(21.16) |  |  | 2198(51.93) | 111(10.11) |  |
| 40-59 | 1103(34.09) | 1220(40.25) |  |  | 1103(34.09) | 418(33.74) |  |
| ≥60 | 561(13.98) | 1352(38.59) |  |  | 561(13.98) | 788(56.15) |  |
| **Ethnicity (n, %)** |  |  | 0.03 |  |  |  | <0.001 |
| Non-Hispanic white | 1722(69.50) | 1404(68.70) |  |  | 1722(69.50) | 441(60.06) |  |
| Non-Hispanic black | 747(9.94) | 751(11.61) |  |  | 747(9.94) | 398(17.28) |  |
| Mexican American | 573(7.23) | 533(7.74) |  |  | 573(7.23) | 228(9.04) |  |
| Others | 820(13.34) | 662(11.95) |  |  | 820(13.34) | 250(13.62) |  |
| **Educational level (n, %)**^3^ |  |  | <0.001 |  |  |  | <0.001 |
| ≤12 years | 560(9.89) | 759(14.60) |  |  | 560(9.89) | 433(24.49) |  |
| ＞12 years | 2981(85.95) | 2503(84.20) |  |  | 2981(85.95) | 875(75.24) |  |
| missing | 321(4.17) | 88(1.20) |  |  | 321(4.17) | 9(0.27) |  |
| **Poverty-income ratio (n, %)**^4^ |  |  | <0.001 |  |  |  | <0.001 |
| ≤1.30 | 1150(20.07) | 988(19.88) |  |  | 1150(20.07) | 448(25.09) |  |
| 1.30-3.50 | 1307(32.18) | 1169(34.85) |  |  | 1307(32.18) | 500(41.52) |  |
| >3.50 | 1121(41.93) | 870(37.00) |  |  | 1121(41.93) | 234(25.87) |  |
| missing | 284(5.82) | 323(8.28) |  |  | 284(5.82) | 135(7.52) |  |
| **Body weight (kg)**  **Age: body weight** | 71.28±0.40  0.024±0.00026 | 80.30±0.59  0.029±0.00038 | <0.001  <0.001 |  | 71.28±0.40  0.024±0.00026 | 89.59±0.89  0.030±0.00044 | <0.001  <0.001 |
| **Dietary variables (g)** |  |  |  |  |  |  |  |
| Total monounsaturated fatty acids | 24.73±0.21 | 24.31±0.29 | <0.001 |  | 24.73±0.21 | 23.95±0.58 | <0.001 |
| Total polyunsaturated fatty acids | 16.44±0.16 | 16.18±0.19 | <0.001 |  | 16.44±0.16 | 16.05±0.40 | <0.001 |
| MFA 16:1 (Hexadecenoic)(mg) | 946.52±9.19 | 955.49±14.21 | <0.001 |  | 946.52±9.19 | 943.42±22.76 | <0.001 |
| MFA 18:1 (Octadecenoic) | 23060.06±195.92 | 22680.29±271.58 | <0.001 |  | 23060.06±195.92 | 22339.95±550.58 | <0.001 |
| MFA 20:1 (Eicosenoic) | 245.79±3.64 | 243.33±4.53 | <0.001 |  | 245.79±3.64 | 242.56±6.36 | <0.001 |
| MFA 22:1 (Docosenoic) | 23.65±0.92 | 25.04±1.48 | <0.001 |  | 23.65±0.92 | 26.89±2.69 | <0.001 |
| PFA 18:2 (Octadecadienoic) | 14517.69±142.70 | 14269.81±169.43 | <0.001 |  | 14517.69±142.70 | 14162.90±357.13 | <0.001 |
| PFA 18:3 (Octadecatrienoic) | 1537.09±23.81 | 1532.53±22.13 | <0.001 |  | 1537.09±23.81 | 1501.24±40.02 | <0.001 |
| PFA 18:4 (Octadecatetraenoic) | 10.46±0.52 | 9.40±0.60 | <0.001 |  | 10.46±0.52 | 9.75±0.81 | <0.001 |
| PFA 20:4 (Eicosatetraenoic) | 117.13±1.84 | 120.07±2.02 | <0.001 |  | 117.13±1.84 | 129.26±3.89 | <0.001 |
| PFA 20:5 (Eicosapentaenoic) | 32.34±1.69 | 30.26±1.79 | <0.001 |  | 32.34±1.69 | 28.48±2.53 | <0.001 |
| PFA 22:5 (Docosapentaenoic) | 18.92±0.50 | 19.74±0.64 | <0.001 |  | 18.92±0.50 | 18.55±0.80 | <0.001 |
| PFA 22:6 (Docosahexaenoic) | 61.22±2.60 | 61.49±2.85 | <0.001 |  | 61.22±2.60 | 57.43±3.86 | <0.001 |
| Total energy intake (kcal/day) | 1822.06±11.60 | 1768.57±14.29 | <0.001 |  | 1822.06±11.60 | 1671.79±28.62 | <0.001 |
| **Fast plasma glucose (mg/dL)** | 90.62±0.14 | 104.48±0.20 | <0.001 |  | 90.62±0.14 | 153.40±1.94 | <0.001 |
| **Hemoglobin A1c (%)** | 5.19±0.0060 | 5.62±0.0079 | <0.001 |  | 5.19±0.0060 | 7.18±0.055 | <0.001 |
| **Insulin (Μu/Ml)** | 9.20±0.21 | 14.38±0.33 | <0.001 |  | 9.20±0.21 | 20.08±0.73 | <0.001 |

^1^ Values are given as mean±SE for continuous variables, or frequencies for categorical variables. The significant differences between groups were tested using Multivariable logistic regression analyses tests for continuous variables, and Rao-Scott modified chi-square tests for categorical variables.

^2^ Type 2 diabetes was defined as FPG≥126 mg/Dl or HbA1c≥6.5%, or self-reported use of insulin or oral agents. Prediabetes was defined as FPG 100-125 mg/Dl or HbA1c 5.7%-6.5%(22).

^3^ Over 12 years of school education.

^4^ The ratio is the total family income divided by the poverty threshold

**Table S5.** Characteristics^1^ stratified by tertiles of fatty acid intake for participants in NHANES 2005-2020.3(n = 16,290).

|  | **Total sample** | **MUFA(g/d)** | | | ***p* value** | **PUFA (g/d)** | | | ***p* value** |
| --- | --- | --- | --- | --- | --- | --- | --- | --- | --- |
|  |  | **T1** | **T2** | **T3** |  | **T1** | **T2** | **T3** |  |
| **n** |  | 14.93$\pm$0.07 | 25.52$\pm$0.06 | 43.47$\pm$0.20 | <0.001 | 9.03$\pm$0.04 | 16.09$\pm$0.04 | 28.39$\pm$0.15 | <0.001 |
| **Sex** |  |  |  |  | <0.001 |  |  |  | <0.001 |
| Male (n, %) | 7761 (47.60) | 1743 (28.56) | 2504 (44.17) | 3514 (66.68) |  | 1956 (33.31) | 2585 (45.48) | 3220 (61.35) |  |
| Female | 8529 (52.40) | 3687 (71.44) | 2926 (55.83) | 1916 (33.32) |  | 3474 (66.69) | 2845 (54.52) | 2210 (38.65) |  |
| **Age (years)** |  |  |  |  | <0.001 |  |  |  | <0.001 |
| 18-39 | 5797 (35.70) | 1674 (33.20) | 1917 (35.54) | 2206 (37.92) |  | 1728 (33.51) | 1928 (35.86) | 2141 (37.35) |  |
| 40-59 | 5175 (36.96) | 1591 (34.03) | 1723 (36.15) | 1861 (40.15) |  | 1566 (34.48) | 1748 (36.58) | 1861 (39.35) |  |
| ≥60 | 5318 (27.35) | 2165 (32.76) | 1790 (28.31) | 1363 (21.93) |  | 2136 (32.02) | 1754 (27.56) | 1428 (23.30) |  |
| **Ethnicity (n, %)** |  |  |  |  | <0.001 |  |  |  | <0.001 |
| Non-Hispanic white | 7010 (68.80) | 2140 (65.40) | 2372 (69.51) | 2498 (70.97) |  | 2242 (66.54) | 2384 (69.55) | 2384 (69.97) |  |
| Non-Hispanic black | 3511 (10.46) | 1104 (10.50) | 1165 (10.38) | 1242 (10.49) |  | 1062 (10.22) | 1116 (9.79) | 1333 (11.27) |  |
| Mexican American | 2522 (8.16) | 834 (8.17) | 857 (8.03) | 831 (8.28) |  | 892 (8.58) | 844 (7.97) | 786 (7.99) |  |
| Others | 3247 (12.58) | 1352 (15.94) | 1036 (12.08) | 859 (10.27) |  | 1234(14.66) | 1086 (12.68) | 927 (10.78) |  |
| **High educational level (n, %)**^2^ |  |  |  |  | <0.001 |  |  |  | <0.001 |
| ≤12 years | 3494 (14.61) | 1449 (18.05) | 1116 (13.85) | 929 (12.47) |  | 1492 (19.11) | 1095 (13.40) | 907 (11.91) |  |
| ＞12 years | 11908 (85.39) | 3730 (81.95) | 4021 (86.15) | 4157 (87.53) |  | 3699 (80.89) | 4057 (86.60) | 4152 (88.09) |  |
| **Poverty-income ratio (n, %)**^3^ |  |  |  |  | <0.001 |  |  |  | <0.001 |
| ≤1.30 | 4596 (20.23) | 1732 (25.54) | 1514 (19.09) | 1350 (16.97) |  | 1767 (25.25) | 1445 (18.69) | 1384 (17.46) |  |
| 1.30-3.50 | 5698 (36.27) | 1876 (36.78) | 1915 (37.43) | 1907 (34.77) |  | 1932 (38.19) | 1930 (36.96) | 1836 (33.98) |  |
| >3.50 | 4584 (43.50) | 1268 (37.68) | 1547 (43.48) | 1769 (48.26) |  | 1218 (36.55) | 1595 (44.35) | 1771 (48.56) |  |
| **Total energy intake** (Kcal/day) | 2086.55$\pm$8.94 | 1383.92$\pm$7.12 | 1957.37$\pm$7.70 | 2792.82$\pm$12.87 | <0.001 | 1462.60$\pm$8.46 | 1982.72$\pm$10.02 | 2696.83$\pm$13.41 | <0.001 |
| **Body weight** (kg) | 83.04$\pm$0.24 | 79.19$\pm$0.42 | 82.10$\pm$0.38 | 87.07$\pm$0.43 | <0.001 | 79.19$\pm$0.42 | 82.10$\pm$0.38 | 87.07$\pm$0.44 | <0.001 |
| **Fast plasma glucose (**mg/dL**)** | 106.32±0.37 | 105.70±0.54 | 105.87±0.65 | 107.27±0.56 | <0.001 | 106.00±0.51 | 105.71±0.59 | 107.15±0.57 | <0.001 |
| **Hemoglobin A1c (%)** | 5.61±0.012 | 5.63±0.019 | 5.61±0.018 | 5.61±0.018 | <0.001 | 5.62±0.018 | 5.60±0.018 | 5.62±0.018 | <0.001 |
| **Insulin (μU/mL)** | 12.84$\pm$0.18 | 12.06$\pm$0.24 | 12.57$\pm$0.23 | 13.72$\pm$0.39 | <0.001 | 12.48$\pm$0.25 | 12.45$\pm$0.21 | 13.59$\pm$0.45 | <0.001 |
| **Diabetes mellitus (n, %)**^4^ |  |  |  |  | <0.001 |  |  |  | <0.001 |
| Prediabetes | 7153 (44.34) | 2266 (41.17) | 2351 (43.01) | 2536 (48.23) |  | 2450 (46.43) | 2423 (44.77) | 2280 (42.14) |  |
| Type 2 diabetes | 2772 (13.16) | 1038 (14.40) | 913 (13.07) | 821 (12.22) |  | 1199 (17.88) | 912 (12.84) | 661 (9.43) |  |

^1^Values are given as mean±SE for continuous variables, or frequencies for categorical variables. The significant differences between tertiles were tested using multivariable logistic regression analyses tests for continuous variables, and Rao-Scott modified chi-square tests for categorical variables.

^2^Type 2 diabetes was defined as FPG≥126 mg/dL or HbA1c≥6.5%, or self-reported use of insulin or oral agents. Prediabetes was defined as FPG 100-125 mg/dL or HbA1c 5.7%-6.5% (22).


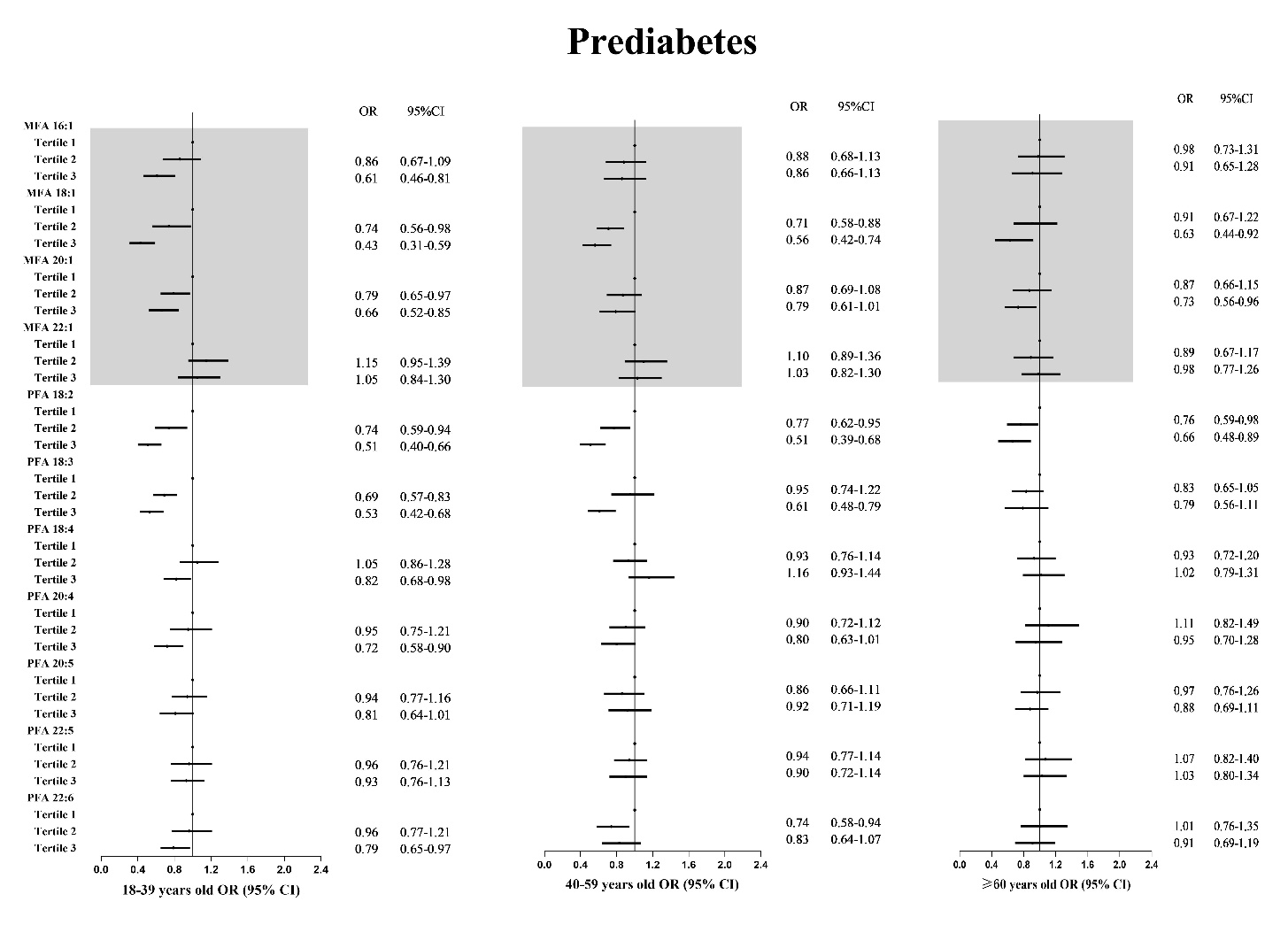


**Figure S1.** Odds ratios with 95% confidence intervals for odds for prediabetes and specific types of monounsaturated fatty acids and polyunsaturated fatty acids for participants in NHANES stratified by age. MUFA= total monounsaturated fatty acids, PUFA= total polyunsaturated fatty acids, OR= Odds ratios, CI= confidence intervals. OR and 95% CI were adjusted by adjusted for gender, age, ethnicity, educational level, poverty-income ratio, and total energy intake.


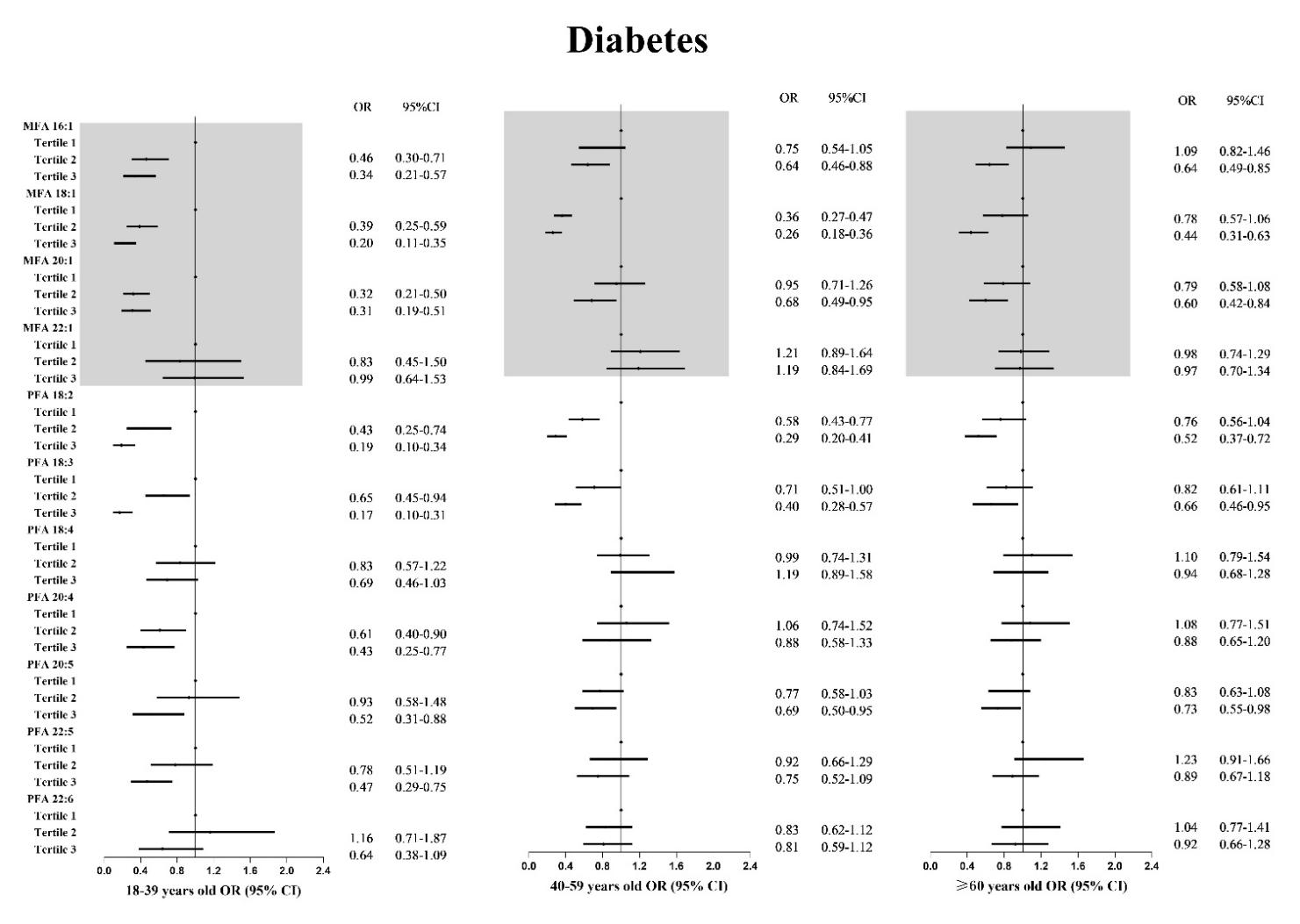


**Figure S2.** Odds ratios with 95% confidence intervals for odds for diabetes and specific types of MUFA and PUFA for participants in NHANES stratified by age. MUFA= total monounsaturated fatty acids, PUFA= total polyunsaturated fatty acids, OR= Odds ratios, CI= confidence intervals.OR and 95% CI were adjusted by adjusted for gender, age, ethnicity, educational level, poverty-income ratio, and total energy intake.

**Table S6.** Total saturated fatty acids, total monounsaturated fatty acids and total polyunsaturated fatty acids intake of fast plasma glucose (mg/dL) for participants in NHANES 2005-2020.3 **^1^.**

|  |  | **Fast plasma glucose** |  |
| --- | --- | --- | --- |
|  |  | Estimate (SE) (mg/d) | *P* value |
| MFA 16:1**^2^** | | -0.23 (0.044) | <0.001 |
| MFA 18:1 | | -0.02 (0.0027) | <0.001 |
| MFA 20:1 | | -0.51 (0.11) | <0.001 |
| MFA 22:1 | | -0.20 (0.16) | 0.23 |
| PFA 18:2 | | -0.024 (0.0034) | <0.001 |
| PFA 18:3 | | -0.15 (0.026) | <0.001 |
| PFA 18:4 | | -2.38 (0.61) | <0.001 |
| PFA 20:4 | | -0.46 (0.35) | 0.19 |
| PFA 20:5 | | -0.71 (0.18) | <0.001 |
| PFA 22:5 | | -2.56 (0.61) | <0.001 |
| PFA 22:6 | | -0.46 (0.10) | <0.001 |

^1^ Values are odds ratio and 95% confidence interval calculated by multinomial logistic regression, after adjusted for gender, age, race, high educational level, poverty-income ratio and total energy intake (Kcal/d).

^2^ The specific unsaturated fatty acids intake was adjusted by weight.

**Table S7.** Total saturated fatty acids, total monounsaturated fatty acids and total polyunsaturated fatty acids intake of HbA1c (%) for participants in NHANES 2005-2020.3 **^1^.**

|  |  | **HbA1c** |  |
| --- | --- | --- | --- |
|  |  | Estimate (SE) (mg/d) | *P* value |
| MFA 16:1**^2^** | | -0.0068 (0.0014) | <0.001 |
| MFA 18:1 | | -0.00057 (0.000085) | <0.001 |
| MFA 20:1 | | -0.0096 (0.0034) | 0.005 |
| MFA 22:1 | | -0.0082 (0.0048) | 0.091 |
| PFA 18:2 | | -0.00059 (0.00012) | <0.001 |
| PFA 18:3 | | -0.0037(0.00071) | <0.001 |
| PFA 18:4 | | -0.050 (0.018) | 0.007 |
| PFA 20:4 | | -0.014 (0.0088) | 0.11 |
| PFA 20:5 | | -0.022 (0.0063) | <0.001 |
| PFA 22:5 | | -0.061 (0.019) | 0.001 |
| PFA 22:6 | | -0.013 (0.0035) | <0.001 |

^1^ Values are odds ratio and 95% confidence interval calculated by multinomial logistic regression, after adjusted for gender, age, race, high educational level, poverty-income ratio and total energy intake (Kcal/d).

^2^ The specific unsaturated fatty acids intake was adjusted by weight.

**Table S8.** Total saturated fatty acids, total monounsaturated fatty acids and total polyunsaturated fatty acids intake of fast plasma glucose (mg/dL) for participants in NHANES 2005-2020.3 **^1^.**

|  |  | **Fast plasma glucose** |  |
| --- | --- | --- | --- |
|  |  | Estimate (SE) (mg/d) | *P* value |
| MFA 16:1**^2^** | |  |  |
| 18-39 | | -0.20 (0.046) | <0.001 |
| 40-59 | | -0.22 (0.079) | 0.007 |
| ≥60 | | -0.30 (0.12) | 0.01 |
| MFA 18:1 | |  |  |
| 18-39 | | -0.018 (0.0031) | <0.001 |
| 40-59 | | -0.023 (0.0051) | <0.001 |
| ≥60 | | -0.022 (0.0048) | <0.001 |
| MFA 20:1 | |  |  |
| 18-39 | | -0.53 (0.14) | <0.001 |
| 40-59 | | -0.33 (0.21) | 0.12 |
| ≥60 | | -0.63 (0.18) | <0.001 |
| MFA 22:1 | |  |  |
| 18-39 | | 0.051 (0.44) | 0.91 |
| 40-59 | | 0.063 (0.45) | 0.89 |
| ≥60 | | -0.37 (0.17) | 0.031 |
| PFA 18:2 | |  |  |
| 18-39 | | -0.020 (0.0045) | <0.001 |
| 40-59 | | -0.028 (0.0074) | <0.001 |
| ≥60 | | -0.027 (0.0066) | <0.001 |
| PFA 18:3 | |  |  |
| 18-39 | | -0.15 (0.037) | <0.001 |
| 40-59 | | -0.16 (0.050) | 0.002 |
| ≥60 | | -0.14 (0.039) | <0.001 |
| PFA 18:4 | |  |  |
| 18-39 | | -1.63 (0.74) | 0.03 |
| 40-59 | | -1.95 (0.97) | 0.046 |
| ≥60 | | -3.79 (1.35) | 0.006 |
| PFA 20:4 | |  |  |
| 18-39 | | -0.88 (0.35) | 0.02 |
| 40-59 | | -0.041 (0.80) | 0.96 |
| ≥60 | | -0.44 (0.49) | 0.38 |
| PFA 20:5 | |  |  |
| 18-39 | | -0.37 (0.30) | 0.22 |
| 40-59 | | -0.66 (0.28) | 0.022 |
| ≥60 | | -1.07 (0.21) | <0.001 |
| PFA 22:5 | |  |  |
| 18-39 | | -2.18 (1.12) | 0.054 |
| 40-59 | | -2.11 (0.92) | 0.02 |
| ≥60 | | -3.34 (0.77) | <0.001 |
| PFA 22:6 | |  |  |
| 18-39 | | -0.29 (0.097) | 0.004 |
| 40-59 | | -0.35 (0.19) | 0.07 |
| ≥60 | | -0.39 (0.17) | 0.02 |

^1^ Values are odds ratio and 95% confidence interval calculated by multinomial logistic regression, after adjusted for gender, age, race, high educational level, poverty-income ratio and total energy intake (Kcal/d).

^2^ The specific unsaturated fatty acids intake was adjusted by weight.

**Table S9.** Total saturated fatty acids, total monounsaturated fatty acids and total polyunsaturated fatty acids intake of HbA1c (%) for participants in NHANES 2005-2020.3 **^1^.**

|  |  | **HbA1c** |  |
| --- | --- | --- | --- |
|  |  | Estimate (SE) (mg/d) | *P* value |
| MFA 16:1**^2^** | |  |  |
| 18-39 | | -0.0058 (0.0015) | <0.001 |
| 40-59 | | -0.0086 (0.0023) | <0.001 |
| ≥60 | | -0.0059 (0.0038) | 0.13 |
| MFA 18:1 | |  |  |
| 18-39 | | -0.00049 (0.00011) | <0.001 |
| 40-59 | | -0.00071 (0.00015) | <0.001 |
| ≥60 | | -0.00046 (0.00015) | 0.003 |
| MFA 20:1 | |  |  |
| 18-39 | | -0.014 (0.0048) | 0.005 |
| 40-59 | | -0.0049 (0.0067) | 0.47 |
| ≥60 | | -0.0080 (0.0055) | 0.15 |
| MFA 22:1 | |  |  |
| 18-39 | | 0.010 (0.014) | 0.46 |
| 40-59 | | -0.013 (0.013) | 0.32 |
| ≥60 | | -0.010 (0.0055) | 0.07 |
| PFA 18:2 | |  |  |
| 18-39 | | -0.00057 (0.00017) | <0.001 |
| 40-59 | | -0.00067 (0.00025) | 0.007 |
| ≥60 | | -0.00047 (0.00017) | 0.008 |
| PFA 18:3 | |  |  |
| 18-39 | | -0.0037 (0.0012) | 0.003 |
| 40-59 | | -0.0035 (0.0013) | 0.011 |
| ≥60 | | -0.0036 (0.011) | 0.0017 |
| PFA 18:4 | |  |  |
| 18-39 | | -0.047 (0.025) | 0.058 |
| 40-59 | | -0.064 (0.030) | 0.033 |
| ≥60 | | -0.028 (0.047) | 0.56 |
| PFA 20:4 | |  |  |
| 18-39 | | -0.036 (0.011) | <0.001 |
| 40-59 | | 0.0023 (0.019) | 0.90 |
| ≥60 | | -0.0032 (0.015) | 0.83 |
| PFA 20:5 | |  |  |
| 18-39 | | -0.013 (0.0093) | 0.18 |
| 40-59 | | -0.033 (0.0084) | <0.001 |
| ≥60 | | -0.014 (0.013) | 0.28 |
| PFA 22:5 | |  |  |
| 18-39 | | -0.051 (0.036) | 0.16 |
| 40-59 | | -0.074 (0.030) | 0.015 |
| ≥60 | | -0.046 (0.032) | 0.16 |
| PFA 22:6 | |  |  |
| 18-39 | | -0.011 (0.0059) | 0.075 |
| 40-59 | | -0.017 (0.0055) | 0.002 |
| ≥60 | | -0.0080 (0.0073) | 0.28 |

^1^ Values are odds ratio and 95% confidence interval calculated by multinomial logistic regression, after adjusted for gender, age, race, high educational level, poverty-income ratio and total energy intake (Kcal/d).

^2^ The specific unsaturated fatty acids intake was adjusted by weight.
